# The chromosome-level genome and key genes associated with mud-dwelling behavior and adaptations of hypoxia and noxious environments in loach (*Misgurnus anguillicaudatus*)

**DOI:** 10.1186/s12915-023-01517-1

**Published:** 2023-02-01

**Authors:** Bing Sun, Yuwei Huang, L. Filipe C. Castro, Su Yang, Songqian Huang, Wu Jin, He Zhou, Shigeho Ijiri, Yi Luo, Jian Gao, Xiaojuan Cao

**Affiliations:** 1 College of Fisheries, Engineering Research Center of Green development for Conventional Aquatic Biological Industry in the Yangtze River Economic Belt, Ministry of Education, Huazhong Agricultural University, Postal address: No.1 Shizishan Stress, Hongshan District, Wuhan, 430070 Hubei Province China; 2 Interdisciplinary Centre of Marine and Environmental Research of the University of Porto, 4450-208 Matosinhos, Portugal; 3 Department of Biology, University of Porto, 4450-208 Porto, Portugal; 4 Department of Aquatic Bioscience, Graduate School of Agricultural and Life Sciences, the University of Tokyo, Bunkyo, Tokyo, 113-8657 Japan; 5 Freshwater Fisheries Research Center, Chinese Academy of Fishery Sciences, Wuxi, 214081 Jiangsu China; 6 College of Fisheries and Life Science, Dalian Ocean University, Dalian, 116023 China; 7 Division of Marine Life Sciences, Graduate School of Fisheries Sciences, Hokkaido University, Hakodate, Hokkaido 041-8611 Japan

**Keywords:** *Misgurnus anguillicaudatus*, Chromosome-anchored genome assembly, Biological adaptations, Mud-dwelling behavior, Intestinal air-breathing, Detoxification function

## Abstract

**Background:**

The loach (*Misgurnus anguillicaudatus*), the most widely distributed species of the family Cobitidae, displays a mud-dwelling behavior and intestinal air-breathing, inhabiting the muddy bottom of extensive freshwater habitats. However, lack of high-quality reference genome seriously limits the interpretation of the genetic basis of specialized adaptations of the loach to the adverse environments including but not limited to the extreme water temperature, hypoxic and noxious mud environment.

**Results:**

This study generated a 1.10-Gb high-quality, chromosome-anchored genome assembly, with a contig N50 of 3.83 Mb. Multiple comparative genomic analyses found that proto-oncogene c-Fos (*fos*), a regulator of bone development, is positively selected in loach. Knockout of *fos* (ID: Mis0086400.1) led to severe osteopetrosis and movement difficulties, combined with the comparison results of bone mineral density, supporting the hypothesis that *fos* is associated with loach mud-dwelling behavior. Based on genomic and transcriptomic analysis, we identified two key elements involved in the intestinal air-breathing of loach: a novel gene (ID: mis0158000.1) and heat shock protein beta-1 (*hspb1*). The flavin-containing monooxygenase 5 (*fmo5*) genes, central to xenobiotic metabolism, undergone expansion in loach and were identified as differentially expressed genes in a drug stress trial. A *fmo5*^*−/−*^ (ID: Mis0185930.1) loach displayed liver and intestine injury, indicating the importance of this gene to the adaptation of the loach to the noxious mud.

**Conclusions:**

Our work provides valuable insights into the genetic basis of biological adaptation to adverse environments.

**Supplementary Information:**

The online version contains supplementary material available at 10.1186/s12915-023-01517-1.

## Background

Teleost fish are the most abundant vertebrate taxon, accounting for more than half of the described species [1]. This diversity is mirrored into an ample array of physiological and behavioral phenotypes [2]. Loach (*Misgurnus anguillicaudatus*), the most widely distributed species of the family Cobitidae, inhabits in the bottom of lakes, ponds, and other freshwater areas with humus-rich mud [3]. Unlike general benthic species (e.g., *Carassius auratus* and *Pelteobagrus fulvidraco*), *M. anguillicaudatus* can easily and flexibly dwell in the bottom mud to survive in extreme conditions such as the elevated water temperature amplitude and drought environment [4, 5]. The mud-dwelling behavior exhibited by this species justifies its alternative name, “mud loach” [6].

Mud sediment with abundant organic matter is often in an oxygen-deficient state, which is unpleasant to many fishes [7, 8]. Interestingly, some fish species, such as *M. anguillicaudatus*, *Ophiocephalus argus*, and *Protopterus annectens*, have evolved accessory air-breathing organs (ABOs) to adapt to anoxic environments [9–11]. Specifically, *M. anguillicaudatus* uses multiple ABOs including posterior intestine, skin, and barbel, to do air-breathing to maintain their blood oxygen saturation [12]. *M. anguillicaudatus* has become an appropriate model for investigating the evolution of benthic adaptability in the ABOs [12, 13]. Comparative transcriptome analysis of posterior intestines between *M. anguillicaudatus* and *Leptobotia elongata* (a Cobitidae fish without ABO) has revealed the genetic basis for the enhanced oxygen transportability of hemoglobin genes and the ABO vascularization in air-breathing fish [14]. In hypoxic mud, the humus decomposition easily leads to the accumulation of noxious substances. In addition, the continuous discharge of sewage containing polycyclic aromatic hydrocarbons (PAHs) generated from urban industrialization and human activities makes the benthic mud a greater threat to the survival of all aquatic organisms than before [15, 16]. However, *M. anguillicaudatus* displays a number of adaptive solutions to the adverse mud environment and serves as a valuable model for studying the underlying genetic basis.

Here, we presented the first high-quality, chromosome-anchored genome assembly of the *M. anguillicaudatus*, and subsequently carried out comparative evolutionary and genomic analyses with several other fish genomes to investigate the origins of phenotypic novelty in the loach. Together with transcriptome and CRISPR-Cas9 gene knockout system, we analyzed the behavioral and biological characteristics of *M. anguillicaudatus* in the benthic mud and uncovered the genetic factors resulting in phenotypes including mud-dwelling, intestinal air-breathing, and detoxification. In conclusion, this study provides insights into the adaptations of *M. anguillicaudatus* to adverse environments including extreme water temperature variations and hypoxic and noxious environment.

## Results and discussion

### Genome assembly and annotation

A single male *M. anguillicaudatus* was used for genome sequencing and assembly. A total of 148 Gb of clean sequencing data were generated for the loach using the Illumina HiSeq X Ten platform (Additional file 1: Table S1-(1)). By K-mer (*K* = 17) analysis [17, 18], the genome size of the loach was estimated to be 1135 Mb with 57.60% repeat sequence (Additional file 1: Table S1-(2)).

**Fig. 1:**
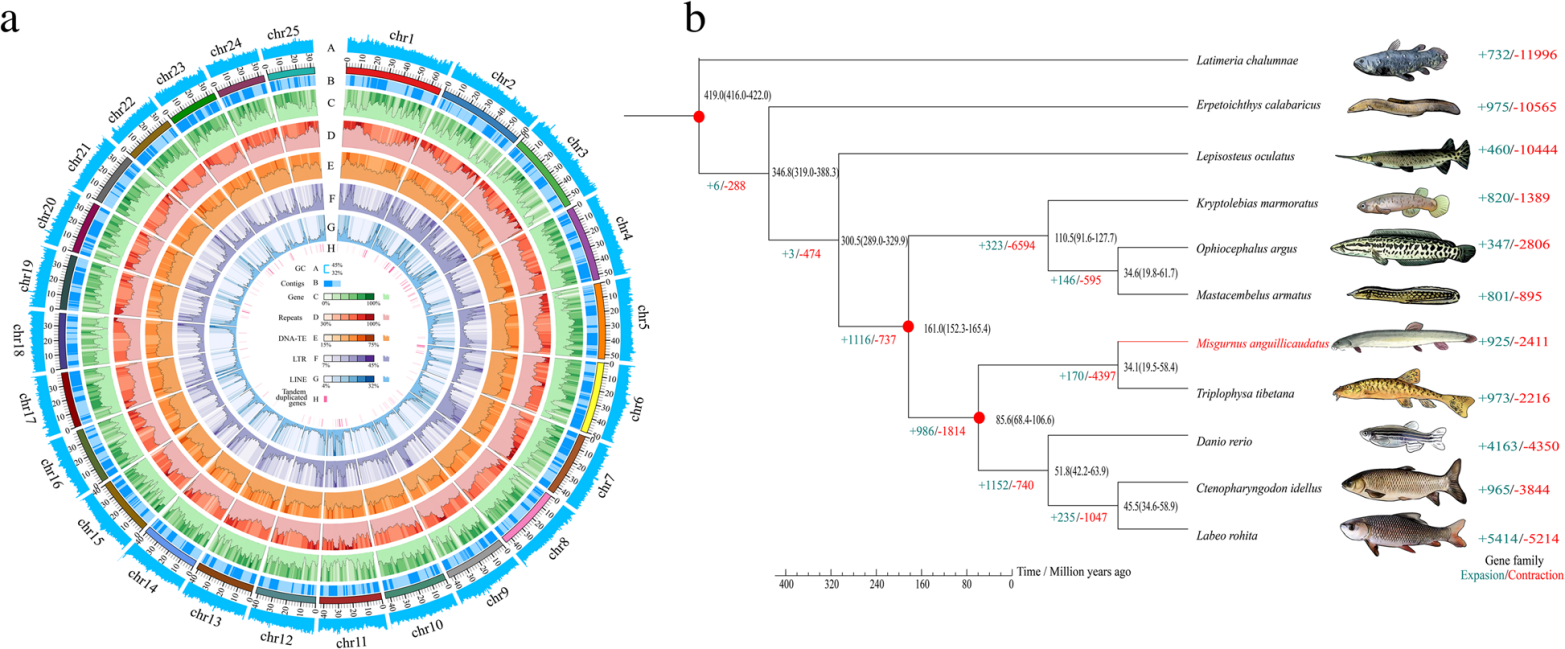
Genome assembly, phylogenomic and gene family change analyses. **a** The Circos plot showing the 25 chromosomes in loach *Misgurnus anguillicaudatus*. Rings (A–H) indicate GC content, contigs, gene density, repeat coverage, DNA-TE (DNA-transposable elements), LTR (long terminal repeats), LINE (long interspersed nuclear elements), and tandem duplicated genes in each pseudo-chromosome of the loach genome. **b** The phylogeny and divergence times of the loach and other fishes. The number in each node represents the divergence time among species and the red circle indicates the fossil record used for calibration in the node. The numbers with slashes represent the expanded and contracted gene families in the node

In addition, we generated 185 Gb long reads by using the PacBio platform to construct the genome of the loach. All corrected PacBio long and short reads were applied for two rounds of assembly corrections. Then, the redundant sequences of the loach genome were removed and the final contig assembly of 1.17 Gb with a contig N50 length of 4.50 Mb was obtained. The genome contained 665 contigs (≥ 100 bp) and 647 contigs (≥ 2 kb), with the longest contigs being 1.70 Mb in length (Table 1). Moreover, by using Hi-C library sequencing, we obtained about 80 Gb of cleaned reads for Hi-C analysis. The assembled genome sequences were anchored to 25 chromosomes with a mounting rate of 98.66% (Fig. 1a; Additional file 1: Table S1-(3) and Additional file 2: Fig. S1). The total assembled length of the loach genome was estimated to be 1.10 Gb, with contig N50 of 3.83 Mb and scaffold N50 of 42.95 Mb, which provided the first chromosomal genome assembly for *M. anguillicaudatus* (Table 1).

**Table 1:** Assembly statistics, BUSCO assessment and Hi-C analysis of loach *Misgurnus anguillicaudatus* genome

Assembly feature		Contig length (bp)	Contig number	Scaffold length (bp)	Scaffold number
N90		1,207,751	267	1,207,751	266
N50		4,463,776	84	4,463,776	84
Total length		1,173,940,613	-	1,173,940,614	-
Number (≥100bp)		-	665	-	664
Number (≥2kb)		-	647	-	646
Max length		1,719,164	-	17,196,164	-
BUSCO assessment	Complete BUSCOs (C)	Complete and single-copy BUSCOs (S)	Complete and duplicated BUSCOs (D)	Fragmented BUSCOs (F)	Missing BUSCOs (M)
Proteins	4325 (94.35%)	3841 (83.79%)	484 (10.56%)	64 (1.40%)	195 (4.25%)
Chromosome-level genome assembly	Number of chromosomes/contigs		Contig N50 (bp)	Scaffold N50 (bp)	Total size
	25/357		3,833,009	42,952,821	1.10 Gb

We found that the loach genome contained 57.35% repetitive sequences, which was equivalent to our estimation from the K-mer method. In the loach genome, DNA transposons (39.39%), long terminal repeats (LTRs, 14.23%), and long interspersed nuclear elements (LINEs, 7.02%) were the most abundant (Additional files 1 and 2: Table S1-(4) and Fig S2a).

The content of repetitive sequences in the loach genome was much higher than that of some fish species, including *Triplophysa tibetana* (39.80%) [19], *Beaufortia kweichowensis* (22.24%) [20], and *Ctenopharyngodon idellus* (38%) [21].

De novo, homology-based prediction and transcriptome sequencing-based methods were used to annotate protein-coding genes in the loach genome. We identified 24,974 protein-coding genes with an average gene length of 22,414 bp (Additional file 1: Table S1-(5)). The distributions of gene length, coding sequences (CDS) length, and exon and intron lengths in the loach genome were similar to those of other six closely related species (*C. auratus*, *Cyprinus carpio*, *Danio rerio*, *Sinocyclocheilus graham*, *S. rhinocerous* and *T. tibetana*) (Additional file 2: Fig. S2b). Gene functional annotation is essential to elucidate gene function and aid further analysis. About 97.29% of the protein-coding genes could be annotated in at least one public database (Additional file 1: Table S1-(6)). In addition, the completeness of the loach genome was further assessed by using Benchmarking universal single-copy orthologues (BUSCO, actinopterygii_odb9 database) (v 3.0) [22]. We detected 94.35% and 1.40% of the completed and fragmented genes of the total of 4325 BUSCO genes in the genome, respectively (Table 1). The number of protein-coding genes in the loach genome was much the same as that in *T*. *tibetana* genome (24,372) [19]. These results indicated that we assembled a high-quality chromosome-level loach genome.

### Phylogenetic relationships

The phylogenetic relationships reconstructed by using 1818 single-copy orthologues from 11 fish species confirmed that *M. anguillicaudatus* is close to the family Cyprinidae and its closest sister species is *T. tibetana*. The time of divergence of *M. anguillicaudatus* and *T. tibetana* was estimated to be 34.10 million years ago (Ma) (Fig. 1b). Moreover, gene family analysis indicated that there were 925 expanded and 2411 contracted gene families in *M. anguillicaudatus* when compared with its most recent common ancestor (MRCA) (Fig. 1b). Meanwhile, 120 genes that appeared to be under positive selection in *M. anguillicaudatus* were identified (false discovery rate (FDR) <0.01). These changes in *M. anguillicaudatus* indicate an essential role in the evolution.

### Alterations in fos (ID: Mis0086400.1) associated with the mud-dwelling behavior

Great changes in the hydrologic environment result in the loss of suitable habitats, ultimately having adverse impacts on the survival of aquatic organisms. *M. anguillicaudatus*, a species distributed in the bottom of extensive freshwater areas, can burrow into the mud to survive from adverse environments [4, 5]. Numerous studies have demonstrated that bone and muscle are vital for most animal locomotion [23–25]. Moreover, the presence of limb bones or the gain of limb-like genes in some fish enhances their adaptability on land [26–28].

To address the genetic basis of the special biological behavior (mud-dwelling) of *M. anguillicaudatus*, we first analyzed the functional annotations of the genes with potential roles in bone or muscle development that expanded or were positively selected in the loach genome. We then identified several genes including expanded myosin complex genes (GO:0016459, *P* = 1.55E−16) and a positively selected gene (PSG) (namely, proto-oncogene c-Fos (*fos*)) involved in osteoclast differentiation in the loach genome (Additional file 1: Table S2-(1 and 2)).

Many studies have demonstrated that myosin plays a central role in muscle contraction [29, 30]. The significant expansion of myosin in *M. anguillicaudatus* therefore suggested its important role in the capability of locomotion. Fos, a primary factor that directs osteoclast differentiation and bone remodeling, is identified in mice [31, 32]. An overexpression of *fos* in mice caused osteosarcomas and bone abnormalities [33]. The deficiency of *fos* led to severe osteopetrosis in mice [ 34, 35]. Together, these have demonstrated that *fos* plays an essential role in bone development.

**Fig. 2:**
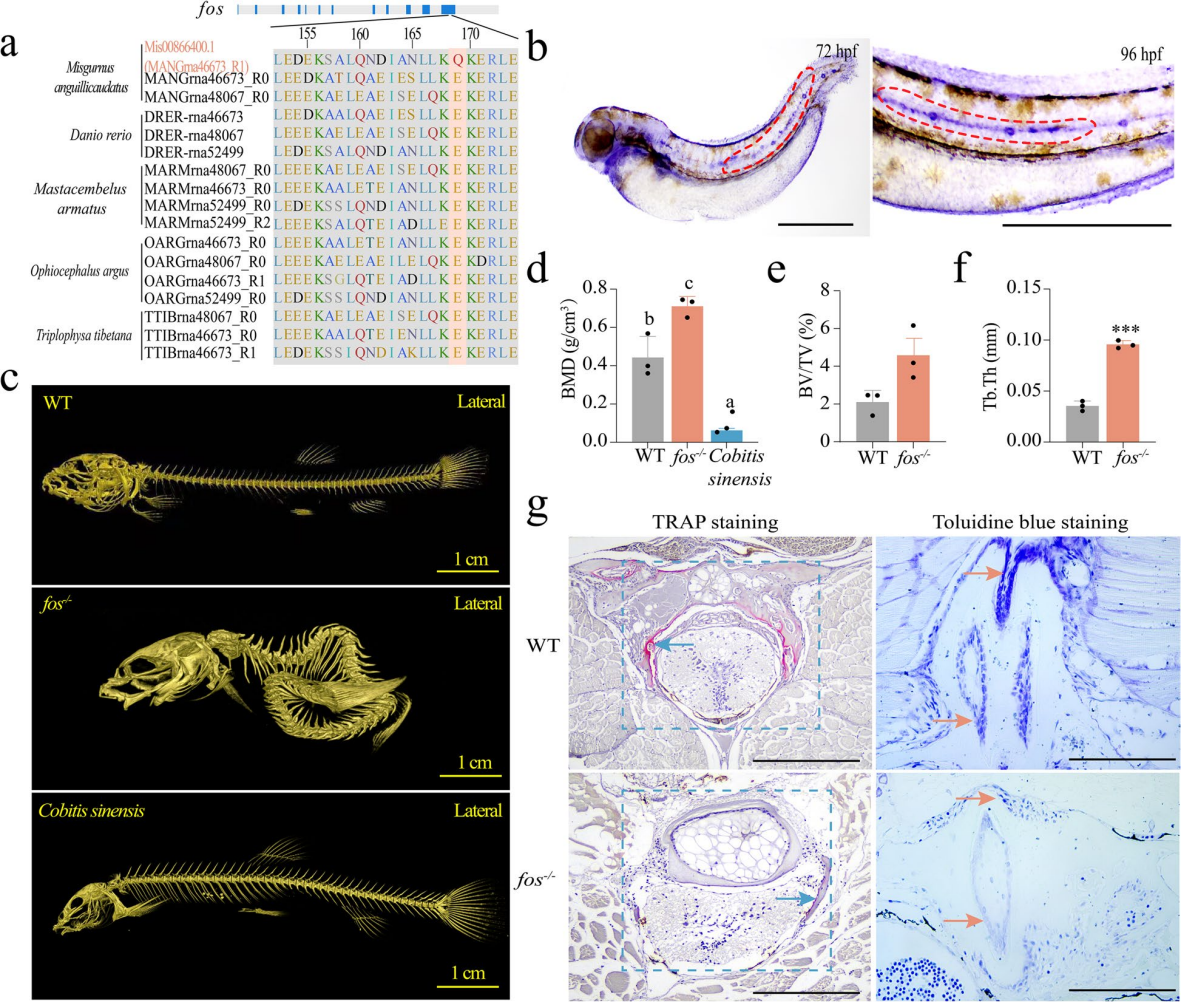
Alterations in *fos* (ID: Mis0086400.1) associated with the mud-dwelling ability of loach *Misgurnus anguillicaudatus*. **a** Alignment of the protein sequences of *fos* from the five species: *M. anguillicaudatus*, *Danio rerio*, *Mastacembelus armatus*, *Ophiocephalus argus*, and *Triplophysa tibetana*. The gray bar with blue stripes shows the exon-intron structure of *fos* and the red word (Q) with salmon color back ground indicates the specific mutation in the *fos* gene of the loach. **b** Whole-mount in situ hybridization (WISH) analysis of *fos*. Red dotted areas indicate the expression signals. hpf, hours post-fertilization. **c** Micro-computed tomography (Micro-CT) analysis of wild-type loach (WT), *fos*-deletion loach (*fos*^*−/−*^), and *Cobitis sinensis*. **d–f** The parameters of spine bones: BMD, BV/TV and Tb.Th. BMD, bone mineral density; BV/TV, bone volume over total volume; Tb.Th, trabecular thickness. **g** Trap staining and toluidine blue staining of spines from WT and *fos*^*−/−*^ loach. Blue arrows indicate the osteoclasts; the salmon color arrows indicate the chondrocytes. *fos*, Proto-oncogene c-Fos. Different letters above error bars indicate significant difference (*p* < 0.05). *** extremely significant difference (*p* < 0.001)

Thus, we identified *fos* gene repertoire in five fish species: *M. anguillicaudatus*—three, *O. argus*—four, *Mastacembelus armatus*—four, *D. rerio*—three, and *T. tibetana*—three (Fig. 2a and Additional file 2: Fig. S3a). As for *M. anguillicaudatus*, a *fos* gene (ID: Mis0086400.1) was positively selected and an amino acid change (at the 12th exon) was identified between *M. anguillicaudatus* and the other four fish species (Fig. 2a). In addition, we found *fos* was highly expressed in the spine of the loach at 72 h post-fertilization (hpf) and 96 hpf (Fig. 2b). Subsequently, we generated a *fos* knockout mutant (four bases (TTGA) missed at the 12th exon of the *fos* gene (ID: Mis0086400.1)) by CRISPR-Cas9, which showed a high mortality rate during their early development (Additional file 2: Fig. S3b-d). Compared with wild-type loach (WT loach), *fos*^*−/−*^ loach showed difficulty in movement (losing the ability of mud-dwelling). Spinal deformity, significantly higher bone mineral density (BMD) and trabecular thickness (Tb.Th), and increased bone volume over total volume (BV/TV) were observed in *fos*^*−/−*^ loach relative to WT loach (Fig. 2c–f). In addition, compared with *Cobitis sinensis* (a benthic loach without mud-dwelling behavior), WT loach *M. anguillicaudatus* presented a significantly higher BMD. Thus, an appropriate BMD might be an important factor for the acquisition of the loach mud-dwelling ability. Furthermore, the number of osteoblasts and chondrocytes in the spine of *fos*^*−/−*^ loach was obviously smaller than that of WT loach (Fig. 2g).

These findings were consistent with the results reported in *fos*-deficiency mice, which exhibited severe osteopetrosis (high BMD) and reduction of osteoblasts and chondrocytes [32, 36]. In this study, the deletion of *fos* in the loach contributed to a significantly higher BMD and spinal deformity, eventually affecting its mud-dwelling ability. It suggests that *fos* would be closely related to the mud-dwelling behavior in the loach.

### Air-breathing and evolution of loach intestine

Oxygen is vital for the survival of most life forms. With multiple ABOs, *M. anguillicaudatus* obviously enhances its hypoxic tolerance, providing a good model for studying the evolution of benthic adaptability in the accessory respiratory organs [12, 14, 37].

**Fig. 3:**
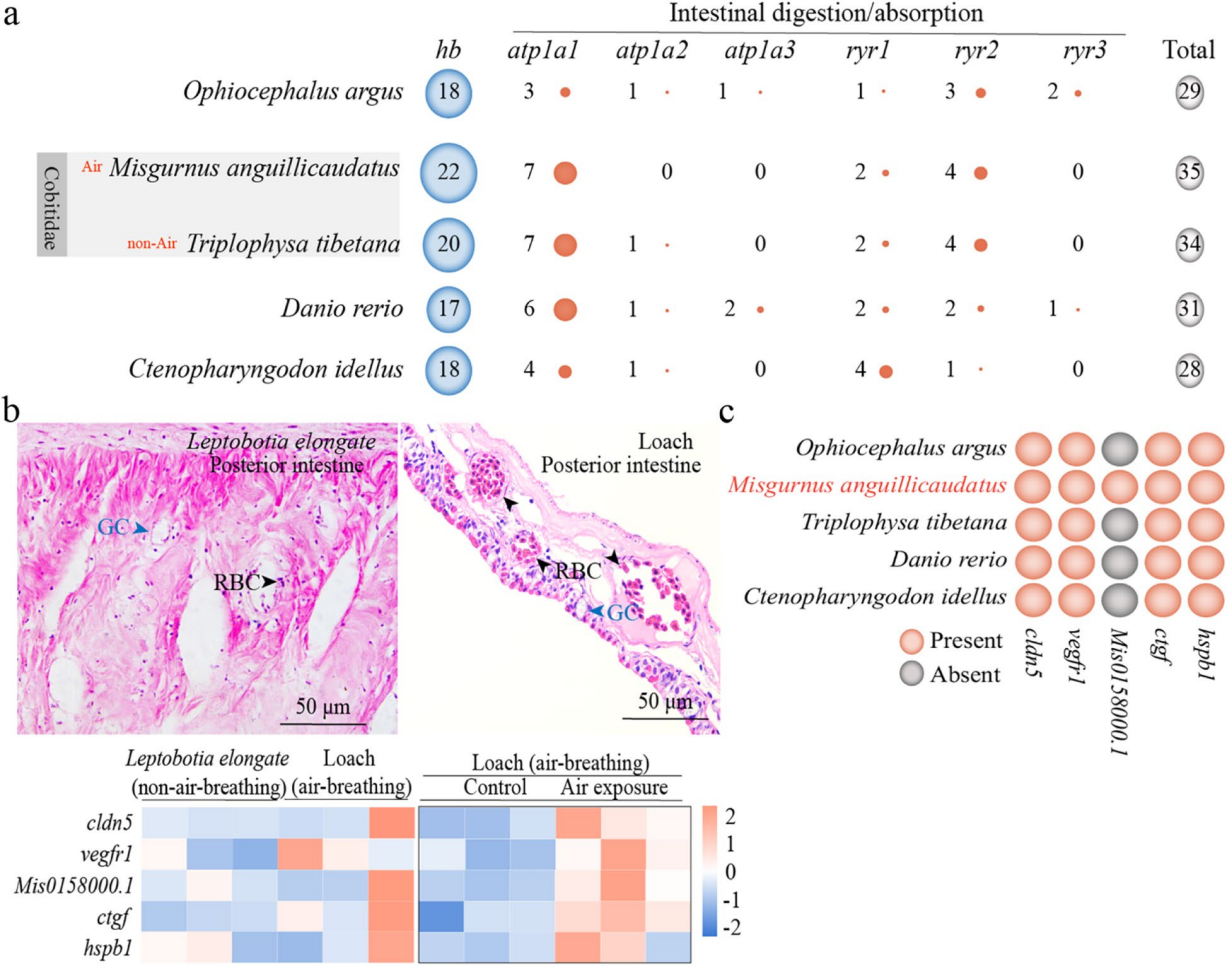
Genes related to mediate intestinal air-breathing and digestion/absorption in fish. **a** The number of hemoglobin (*hb*) gene family and digestion/absorption-related gene families shown in the blue circle and salmon color circle, respectively. The total number of *hb* gene family and digestion/absorption-related gene families is shown in the gray circle. The circle sizes are equivalent to the gene number that was observed. Both *Misgurnus anguillicaudatus* and *Triplophysa tibetana* belong to Cobitidae. Loach *M. anguillicaudatus* is an air-breathing fish, while *T. tibetana* is a non-air-breathing fish*.* **b** The structures of posterior intestines of *Leptobotia elongate* (non-air-breathing fish) and the loach, and the expression profiles of five air-breathing-related genes (namely five DEGs) in the posterior intestines of *L. elongate* and the loach. The blue triangles represent goblet cells (GC for short) and the black triangles represent the red blood cells(RBC for short). The black box represents expression profiles of DEGs in the posterior intestines of loach between the control and air exposure group. **c** The presence and absence of the five DEGs in five fish species. DEGs, differentially expressed genes. *Cldn5*, claudin-5; v*egfr1*, vascular endothelial growth factor receptor1; Mis0158000.1, which is a new gene and annotated as interleukin 1 beta (*il1b*); *ctgf*, connective tissue growth factor; *hspb1*, heat shock protein beta-1

Here, we found that oxygen transport-related GO categories (oxygen binding (GO:0019825, *P* = 3.11E-12), gas transport (GO:0015669, *P* = 1.48E−12), oxygen transport (GO:0015671, *P* = 1.48E−12), and hemoglobin complex (GO:0005833, *P* = 2.86E−13)) have significantly expanded in the loach genome (Additional file 1: Table S2-(3))*.* A total of 22 hemoglobin (*hb*) genes were identified in *M. anguillicaudatus*, which was more than those in the other four fish species (*D. rerio* (17), *O. argus* (18), *T. tibetana* (20), and *C. idellus* (18)) (Fig. 3a and Additional file 2: Fig. S4a). Under air exposure, the expression levels of *hbb* (ID: Mis0119270.1) and *hba* (ID: Mis0119300.1) genes in posterior intestines of *M. anguillicaudatus* were significantly increased (Additional file 2: Fig. S4b).

Multiple studies have demonstrated that *hb* genes are involved in blood oxygen transport, mediating an adaptive response to hypoxia [38–41]. These findings provided evidence of the adaptation of *M. anguillicaudatus* to live in the benthic hypoxia environment.

In the loach genome, the VEGF signaling pathway related to angiogenesis (ko04370, *P* = 9.88E−01) was positively selected (Additional file 1: Table S2-(4)). Our previous analyses in the loach transcriptomes and microRNAs have indicated that some angiogenesis-related genes are involved in the intestinal air-breathing [10, 14, 42, 43]. Based on these findings, several potential air-breathing-related genes were screened out, including hypoxia-inducible factor 1-alpha (*hif1a*), vascular endothelial growth factor A-A (*vegfaa*), vascular endothelial growth factor receptor (*vegfr*), dual specificity mitogen-activated protein kinase kinase 2 (*map2k2*), interleukin 1 beta (*il1b*), claudin-5 (*cldn5*), connective tissue growth factor (*ctgf*), and heat shock protein beta-1 (*hspb1*) (Additional file 1: Table S2-(5-7)). To better screen out the key air-breathing-related genes, we further conducted an air exposure trial of the loach and sampled posterior intestines for RNA-seq. Then, the transcriptome data were verified (Additional file 2: Fig. S4c) and analyzed. Compared with the control (C_chang group), a total of 1273 differentially expressed genes (DEGs) (458 upregulated and 815 downregulated) were found under air exposure (T_chang group). Based on the results of this transcriptome analysis and the previous findings in *M. anguillicaudatus*, we identified 5 DEGs (*cldn5*, *vegfr1*, mis0158000.1 (KO_definition: (K04519; interleukin 1 beta (*il1b*)), *ctgf* and *hspb1*) (Additional file 1: Table S2-(8)), which were obviously upregulated in the posterior intestine (an ABO) of *M. anguillicaudatus* (Fig. 3b). Further, we identified the five genes in five fish species (*M. anguillicaudatus*, *O. argus*, *T. tibetana*, *D. rerio* and *C. idellus*) (Fig. 3c and Additional file 2: Fig. S4d). Mis0158000.1 only exists in *M. anguillicaudatus* and *hspb1* is under significant positive selection in the loach genome. These findings suggested that mis0158000.1 and *hspb1* play a key role in the intestinal air-breathing of *M. anguillicaudatus*.

Here, we also investigated genes related to food digestion or absorption and found that most of the genes, which were from the pathways (salivary secretion (ko04970, *P* = 6.50E−10), mineral absorption (ko04978, *P* = 2.17E−09), carbohydrate digestion and absorption (ko04973, *P* = 3.96E−09), pancreatic secretion (ko04972, *P* = 1.05E−07), protein digestion and absorption (ko04974, *P* = 2.67E−07), bile secretion (ko04974, *P* = 1.52E−06), and gastric acid secretion (ko04971, *P* = 2.32E−06)), were significantly contracted (Additional file 1: Table S2-(9)). The genes from these pathways were identified in five fish species (*M. anguillicaudatus*, *O. argus*, *T. tibetana*, *D. rerio* and *C. idellus*) (Additional file 2: Fig. S4e). It was worth noting that the total number of the sodium/potassium-transporting ATPase *atp1a* family genes (*atp1a1*, *atp1a2* and *atp1a3*) and ryanodine receptor *ryr* family genes (*ryr1*, *ryr2* and *ryr3*) in *M. anguillicaudatus* (13 genes) was less than that of *T. tibetana* (14 genes) (Fig. 3a). These findings may suggest that the contraction of these genes in *M. anguillicaudatus* was due to the air-breathing of posterior intestine. In addition, in the pancreatic secretion pathway (ko04972, *P* = 5.50E−01), we found that two ras-related C3 botulinum toxin substrate 1 genes (*rac1*, Mis0219230.1 and Mis0019870.1) expanded (Additional file 1: Table S2-(10)).

Previous research demonstrated that *rac1*, belonging to the small G protein, is a key regulator of the secretion of pancreatic digestive enzyme [44]. Expansion and contraction of gene families potentially provide functional links between genes and their associated characteristics [45]. Thus, the expansion of *hb* family and *rac1* genes and the contraction of *atp1a* and *ryr* families might help *M. anguillicaudatus* to better balance the digestion/absorption and air-breathing ability in intestines.

### Gene family involved in the detoxification function

Although many noxious substances present in the mud through sedimentation, particle adsorption, or harmful decomposition [46], *M. anguillicaudatus* can survive in the muddy bottom of extensive freshwater areas [47]. So, we searched for genes encoding enzymes with a detoxification function in the loach. Flavin-containing monooxygenase (FMO) is an important Phase I enzyme, participating in the catalyzation of a wide variety of xenobiotics that contain nitrogen, sulfur, or phosphorus [48, 49]. UDP-glucuronosyltransferases (UGTs) catalyze the metabolism of numerous endogenous and xenobiotic compounds with glucuronidation [50, 51].

In the loach genome, the KEGG pathways including cytochrome P450 (ko00983, *P* = 1.77E-04), drug metabolism-cytochrome P450 (ko00980, *P* = 1.06E−05) and drug metabolism-other enzymes (ko00982, *P* = 3.60E−18), which were associated with metabolism of xenobiotics, significantly expanded (Additional file 1: Table S3-(1)). Subsequently, we identified the *fmo5* genes and UGT family in five fish species (*M. anguillicaudatus*, *T. tibetana*, *D. rerio*, *Oreochromis mossambicus*, and *P. fulvidraco*) (Additional file 2: Fig. S5a-b). Compared to other four fish species, we found that the genome of *M. anguillicaudatus* had the highest number of *fmo*5 gene.

To explore the role of FMOs and UGTs in the benthic mud adaptation of *M. anguillicaudatus*, drug stress trails were performed. Multiple studies have indicated that PAHs (like Benzoapyrene, 1-Naphthol and Pyrene) are one of the most common pollutants in the muddy bottom [52, 53]. Some of the most highly industrialized and urbanized locations have an extremely high concentration of PAHs of more than 10,000 μg/g in sediment [54, 55]. In addition, the presence of Bisphenol A (BPA) and p-Nitrophenol exposure in bottom water have raised great exposure concern [56, 57].

**Fig. 4:**
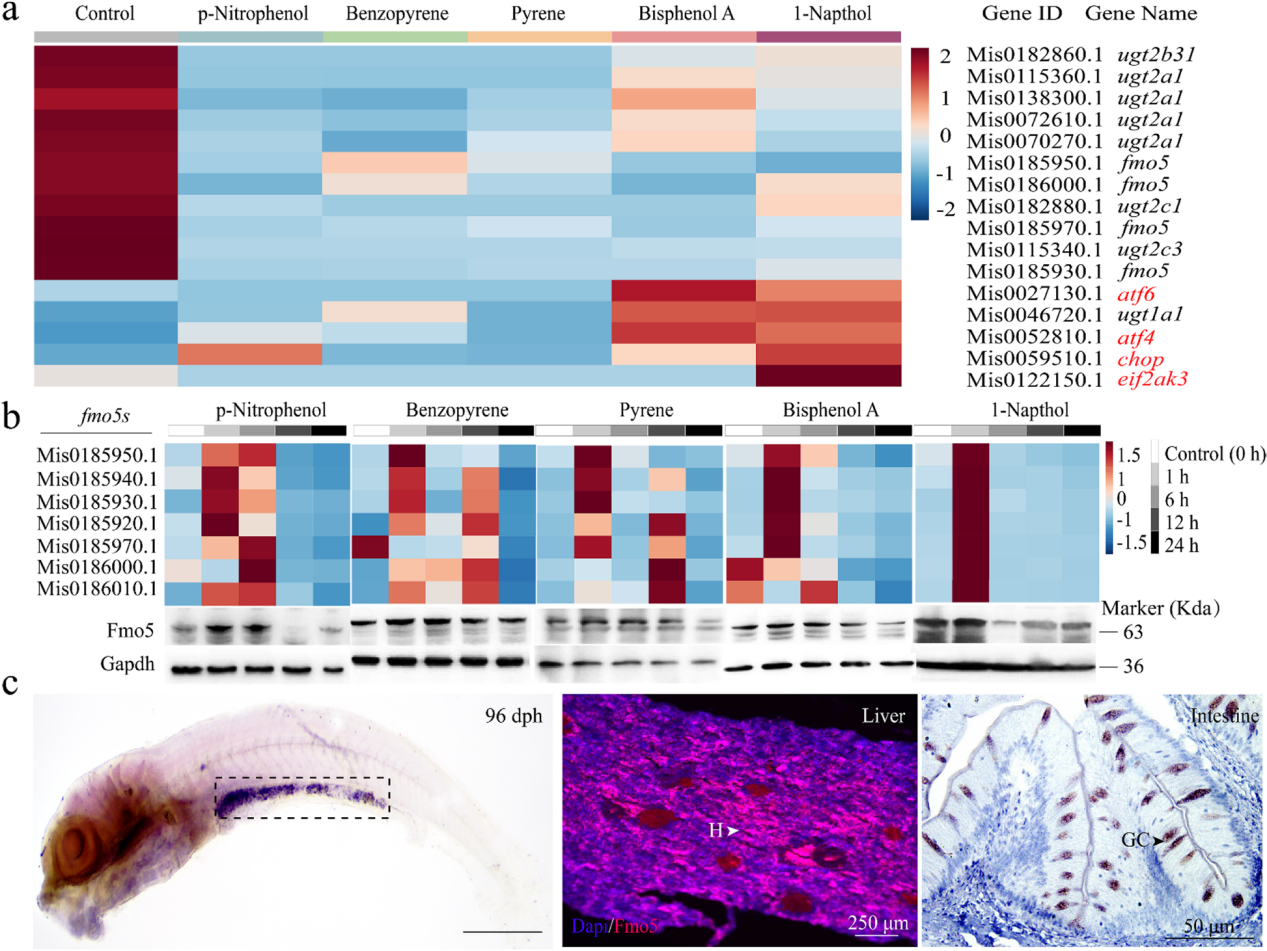
Transcriptome analysis of the drug stress trial and the expression and location of *fmo5* in loach *Misgurnus anguillicaudatus*. **a** The heatmap of several DGEs (namely, *fmo* genes, *ugt* genes, and endoplasmic reticulum stress (RE stress) genes (in red color)) between the control and drug stress group. **b** The expression profiles of *fmo5* genes in livers of wild-type (WT) loach under five drug stress at different time points. **c** Expression and location of *fmo5* gene in the loach. The black dotted box represents the expression signal (liver and intestine tissues) of *fmo5* in the loach (top). Immunofluorescence (lower left; liver tissue) and immunohistochemistry (lower right; intestine tissue) of *fmo5* in the loach. White and black arrows present the expression signal. H, hepatocyte; GC, goblet cell. *Atf6*, cyclic AMP-dependent transcription factor 6; *ugt1a1*, UDP-glucuronosyltransferase 1-1; *eif2ak3*, eukaryotic translation initiation factor 2-alpha kinase 3; *chop*, DNA damage-inducible transcript 3 protein; *atf4*, cyclic AMP-dependent transcription factor 4; Mis0185950.1, Mis0185970.1, Mis0185930.1 (*fmo5*), dimethylaniline monooxygenase [N-oxide-forming] 5; Mis0072610, Mis0115360.1 (*ugt2a1*), UDP-glucuronosyltransferase 2A1

In this study, five drugs (namely, Benzoapyrene, 1-Naphthol, Pyrene, p-Nitrophenol and BPA) were used for stress trails. After 24 h exposure to these drugs, a hepatic transcriptome analysis was performed, and the data were validated (Additional file 2: Fig. S6). The result showed that some DEGs belonging to FMO and UGT families were significantly enriched in the xenobiotic biodegradation and metabolism pathway (Additional file 1: Table S3-(2)), suggesting the involvement of FMO and UGT families in drug metabolism in *M. anguillicaudatus*. However, most of these DEGs were downregulated after 24 h of the treatment (Fig. 4a).

Previous studies have reported that the rate of enzyme activity is related to the concentration of substrate [58]. The downregulation of these DEGs would be linked to the concentrations of these five drugs, which accumulated in liver tissues of the loach. Therefore, we conducted a drug stress trail on WT loach again and the liver tissues were sampled from five different time points (0h, 1 h, 6 h, 12 h, and 24 h) to analyze the gene expression levels.

Results showed that most of the *fmo5* and *ugt* (i.e., *ugt2a1* and *ugt2a2*) genes were obviously upregulated after 1 h treatment with any one of the five drugs (Fig. 4b and Additional file 2: Fig. S7). In addition, after p-Nitrophenol exposure, most of the *fmo5* and *ugt* genes were highly expressed after 6 h treatment. These results further indicated that the *fmo5* and *ugt* genes of *M. anguillicaudatus* participated in the metabolism of these drugs.

In *M. anguillicaudatus*, *fmo5* (ID: Mis0185930.1) was highly expressed in livers and anterior/mid-intestines (located in goblet cells of the intestines) (Fig. 4c and Aditional file 2: Fig. S8a). Liver is the primary organ for detoxification and intestine plays an important role in the immunity of fish [59, 60]. The significant expansion of *fmo* and its high expression in liver and intestine would be essential for the loach to adapt to the noxious bottom mud.

**Fig. 5:**
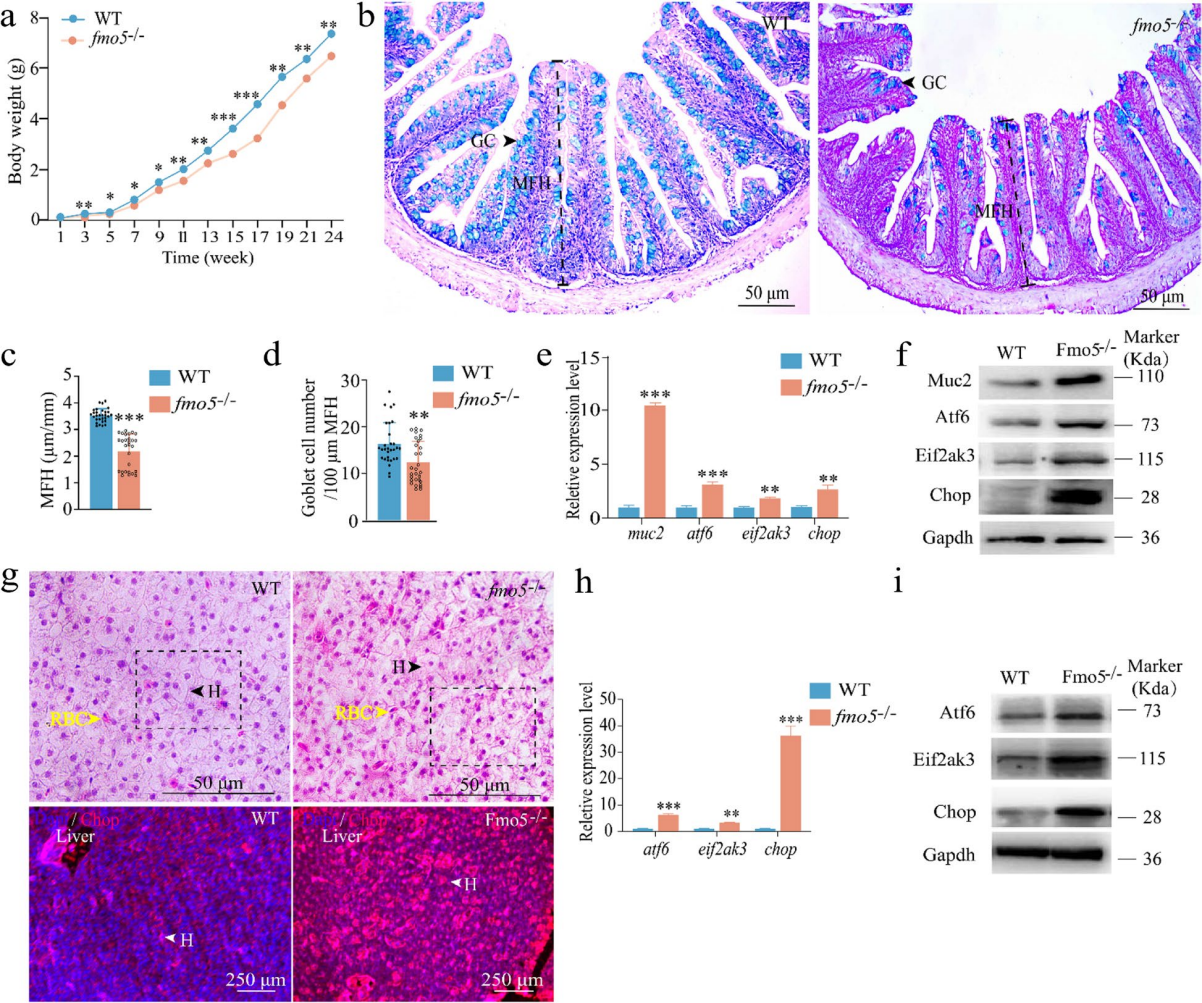
Expansion in *fmo5* (ID: Mis0185930.1) gene involved in the detoxification function of loach *Misgurnus anguillicaudatus*. **a** The growth curves of WT and *fmo5* deletion (*fmo5*^*−/−*^) loach. **b–d** Histological structure analysis of intestines from WT and *fmo5*^*−/−*^ loach. GC, goblet cell; FMH, mucosal fold height. **e,f** Expressions of *muc2* and some endoplasmic reticulum stress (RE stress)-related genes in intestines. **g** Histological structures of livers from WT and *fmo5*^*−/−*^ loach. The black dotted boxes represent the injury area. The black and white triangles represent hepatocyte (H for short). The yellow triangles represent red blood cells (RBC for short). **h,i** Expressions of some RE stress-related genes in livers. *muc2*, Mucin; *atf6*, Cyclic AMP-dependent transcription factor; *eif2ak3*, eukaryotic translation initiation factor 2-alpha kinase 3; *chop*, DNA damage-inducible transcript 3 protein; Gapdh, glyceraldehyde-3-phosphate dehydrogenase. Dapi, 4',6-diamidino-2-phenylindole. * significant difference (*p* < 0.05); ** very significant difference (*p* < 0.01); *** extremely significant difference (*p* < 0.001)

Subsequently, we generated a *fmo5* (ID: Mis0185930.1) knockout mutant (four bases (CGAA) missed at the 3rd exon of *fmo5* gene) by CRISPR-Cas9 (Additional file 2: Fig. S8b and c). *fmo5*^*−/−*^ loach showed significantly slow growth (Fig. 5a). Moreover, we found that the mucosal fold height (MFH) and goblet cell number in the intestine of *fmo5*^*−/−*^ loach were smaller than those of WT loach (Fig. 5b–d).

Similarly, the deletion of *Fmo5* in mice significantly inhibited its growth [61] and reduced the number of goblet cells of colon [62]. The results indicated that the reduction in goblet cell number was due to the high biosynthesis and misfolding of mucin (MUC2) and endoplasmic reticulum stress (ER stress) [62].

We then evaluated the expression levels of *muc2* and ER-stress-related genes (Cyclic AMP-dependent transcription factor (*atf6*), eukaryotic translation initiation factor 2-alpha kinase 3 (*eif2ak3*), and DNA damage-inducible transcript 3 protein (*chop*)). The mRNA and protein expression levels of these genes in intestines of *fmo5*^*−/−*^ loach were higher than those in WT loach (Fig. 5e, f). These findings indicated that the deletion of *fmo5* induced Muc2 expression of intestine, resulting in ER stress and apoptosis in goblet cells, which would affect intestinal functions. Compared with WT loach, an obvious apoptosis occurred in the liver of *fmo5*^*−/−*^ loach (Fig. 5g). We then evaluated the mRNA and protein expression levels of ER stress-related genes in the liver of *fmo5*^*−/−*^ loach and the expression profiles were the same as those in the intestine (Fig. 5h, i). These findings suggested that *fmo5* would be essential for the benthic *M. anguillicaudatus* to maintain health.

Moreover, a drug stress trail of WT loach and *fmo5*^*−/−*^ loach was conducted. Under the treatment of any one of the five drugs, compared with WT loach, *fmo5*^*−/−*^ loach showed an obviously lower survival rate (Additional file 2: Fig. S9), indicating *fmo5*’s critical role in adaptation to adverse environments.

## Conclusions

Advances in genome-sequencing technologies have allowed us to complete the first high-quality, chromosome-anchored genome assembly of *M. anguillicaudatus*. Comparative genomic analyses provided new insights into the genetic basis of vertebrate adaptation to the extreme benthic mud environment. These advancements allow identification of key genes involved in mud-dwelling and intestinal air-breathing and detoxification ability, which will benefit aquaculture and breeding programs. Importantly, considering the increasing global warming impact and worsening environment pollution, the loach genome will be a valuable resource for studying biological adaptations to adverse environments.

## Methods

### Fish maintenance

All diploid loach used in this study were cultured in the recirculating water system with the temperature of 24–26 °C, pH of 7.0–7.5, and dissolved oxygen (DO) of 6.0–6.5 mg/L. Artemia and tubificidae were used to feed loach larvae and loach fingerlings/adults, respectively. The loach was fed three times a day (8:00, 14:00, and 20:00).

### Genome sequencing

The muscle tissues from one diploid male WT loach *M. anguillicaudatus* were used to extract genomic DNA by using the classic phenol-chloroform method. Quality and quantity of the extracted DNA were assessed using the Nanodrop and Qubit, respectively. And the integrity of the extracted DNA was further evaluated on an agarose gel stained with ethidium bromide. The qualified genomic DNA was then sequenced on the platform of PacBio Sequel sequencing (Pacific Biosciences). Genomic DNA of the loach was sheared to an average size using a g-TUBE device (Covaris, Woburn, MA, USA). The sheared DNA was purified and end-repaired using polishing enzymes and then a blunt end ligation reaction followed by exonuclease treatment was applied to create a SMRTbell template according to the PacBio template preparation protocol. After the construction of the library, Qubit 3.0 and Agilent 2100 were used for quantification and detecting the library size, respectively. The library single-molecule sequencing was then conducted on the PacBio Sequel platform to generate long-read data. Moreover, the genome of loach was further sequenced on the Hi-C platform to obtain chromosome-level genome assembly. For Hi-C library construction, DNA extracted from the loach was fragmented and purified using magnetic beads. A Hi-C library (300–350 bp) was constructed and sequenced on the Illumina HiSeq 4000 platform with 150-bp paired-end reads. For RNA sequencing, the complementary DNA libraries were constructed from various tissues according to the manufacturer’s instructions (NEBNext Ultra RNA Library Prep Kit from Illumina, catalog no. E7530S) and sequenced on the Illumina HiSeq 4000 platform (Additional file 1: Table S6).

### Quality control of sequencing data

The different reads including Illumina reads, PacBio reads, and Hi-C reads were quality filtered using different strategies. For Illumina sequencing reads and Hi-C reads, all low-quality reads, duplicated reads, and adapter sequences were removed. For the PacBio reads, the adapter sequences were removed first. Then, any of the reads, which had the content of N exceeding 10% or the number of low-quality base (≤ 5) exceeding 50% of the length of this read, the paired reads were filtered in the single-read sequencing. For Hi-C sequencing data, the low-quality Hi-C reads were filtered by HiC-Pro software [63].

### Genome size estimation

The genome size of the loach was estimated using the K-mer method. The 17-mer was chosen for K-mer analysis in this study, and the genome size (G) was estimated based on the following formula: G = k-mer number/k-mer depth, where k-mer number and k-mer depth represented the total number and the peak of depth of the 17-mer, respectively.

### Genome assembly and chromosome construction

PacBio reads were corrected, trimmed, and assembled using the program Canu (https://github.com/marbl/canu, v 2.1) [64]. The Canu first built read databases (gkpStore) with the settings “min ReadLength = 1,000” and “corOutCoverage = 40”, then built overlap databases (ovlStore) with the settings “minOverlapLength = 500”, and lastly performed an error correction through falcon_sense method (option “correctedErrorRate = 0.025”). Then, Illumina paired-end reads were also aligned to consensus assembly using BWA and the initial draft assembly was polished twice using the Arrow with the setting “miniCoverage = 15”. Pilon (v 1.23) (default parameters) [65] was used to correct the assembled contigs again. According to the redundancy in the assembly results, we subsequently used Purge_haplotigs (https://bitbucket.org/mroachawri/purge_haplotigs.git) (default parameters) to reduce the redundant sequences and obtained the final assembled contigs. Finally, the assembled contigs were corrected mis-joins, orders, orients, and anchored contigs from the draft assembly into a candidate chromosome-length assembly by Hi-C data using Juicer (v 1.5) (default parameters) [66] and 3D-DNA (v 180922) (default parameters) [67]. To further improve the quality and interactive correction, we reviewed the candidate assembly with JuiceBox Assembly Tools (https://github.com/theaidenlab/juicebox). Last, BUSCO (v 4.1.4) was used to estimate the genome quality.

### Genome annotation

Tandem repeats in the loach genome were detected using Tandem Repeats Finder (v 4.09; default parameters) [68]. Repeat elements in the loach genome were annotated using both de novo and homology-based methods. Transport elements (TEs) in the loach genome were identified using RepeatMasker (v 4.0.6) and RepeatProteinMask (v 1.36) with default parameters based on the RepBase database (v 21.12) [69]. For de novo predictions, a de novo transposable element library was constructed using RepeatModeler (v 1.0.5), which was then used to predict repeats with RepeatMasker (v 4.0.6) with default parameters [70].

Protein-coding genes in the loach genome were annotated by combining ab initio predictions, homology-based prediction, and RNA sequencing (RNA-seq)-based methods. For ab initio annotation, Augustus (v 3.2.3) [71] and GENESCAN (v 1.0) [72] were used. For homology-based prediction, the protein sequences of five species (*D. rerio*, *C. auratus*, *C. carpio*, *S. grahami*, and *S. rhinocerous*) were downloaded from NCBI and aligned to the loach genome sequences using TBLASTN (*e*-value less than 1×10^*−*5^). For predicting the protein-coding gene models, Genewise (v 2.4.1) was used to analyze all alignments. For RNA-seq-based method, cleaned RNA-seq reads were assembled into transcripts and then were aligned against the assembled genome to link spliced alignments. Then, results from the three methods were integrated by EVidenceModeler (EVM, v 1.1.1) [73]. Functional annotations of these predicted genes were analyzed using the public function databases. InterProscan (v 4.8) was used to screen proteins against a database (Pfam, v 27.0). Moreover, GO (v 20171220), KEGG (v 89.1), NR (https://www.ncbi.nlm.nih.gov/refseq/about/nonredundantproteins/), Swissprot (uniport: release-201906), and TrEMBL (uniport: release-201906) databases were used for gene functional annotations using BLAST software (v 2.6.0) with *e*-value of 1×10^*−*5^.

### Phylogenetic analysis

Protein sequences of the loach and other 10 fish species (*L. chalumnae*, *E. calabaricus*, *L. oculatus*, *K. marmoratus*, *O. argus*, *M. armatus*, *T. tibetana*, *D. rerio*, *C. idellus*, and *L. rohita*) were analyzed by OrthoMCL (v 2.0.9, default parameters) [74] and the protein-coding genes of the 10 species were downloaded from the NCBI database. All the 1818 one-to-one orthologous genes were identified and aligned. Then, the aligned sequences were concatenated into supergenes used for subsequent analyses. The maximum-likelihood method was used to construct the phylogenetic tree using RAxML (v 8.2.10) [75]. The divergence times of these species were estimated through the Bayesian relaxed molecular clock approach using MCMCtree (v 4.8) in PAML package [76]. Fossil records were obtained from TIMETREE website (http://www.timetree.org) and used for calibrating the calculated divergence time.

### Gene family expansion and contraction

Based on the results in the previous steps (phylogenetic tree and divergence time analysis), the expansion and contraction of gene families were determined using the CAFE software (v 3.1) with a probabilistic model. The *p* value for each gene family was calculated, then *p* value < 0.05 was treated as having a significantly accelerated rate of expansion or contraction. Gene expansion and contraction results for each branch of the phylogenetic tree were estimated, and enrichment analysis about the gene families expanded or contracted in loach was performed with KOBAS (v3.0).

### Detection of PSGs

All one-to-one orthologous gene families from the 11 fish species were extracted to identify PSGs. The high-quality multiple-protein alignments were generated and used to estimate three types of *ω* (the ratio of the rate of non-synonymous substitutions to the rate of synonymous substitutions) using the CodeML program in the PAML package (v 4.8). Branch model (model=2, NSsites=0) was used to detect *ω* of appointed branch to test (*ω*0) and average *ω* of all the other branches (*ω*1) and the mean of whole branches (*ω*2). Then *χ*^2^ test was used to check whether *ω*0 was significantly higher than *ω*1 and *ω*2 under the threshold *p* value < 0.05, which hinted that these genes would be under positive selection or fast evolution.

### Identification of specific amino acid mutations of fos

Genomes of the loach and other four fish species (*O. argus*, *M. armatus*, *T. tibetana*, and *D. rerio*) were used for the analysis of *fos* genes. We collected the genome sequences of *O. argus*, *M. armatus*, and *T. tibetana* from NCBI (http://www.ensembl.org) for comparative analysis. To identify full complement *fos* genes in the genomes, the Fos protein sequences of zebrafish from ZFIN database (http://zfin.org/) were collected and used as queries to conduct TBLASTN (with *e*-value of 1×10^*−*5^) searches against each of the genomes. Gene annotations for the zebrafish *fos* gene clusters are shown in Additional file 1: Table S4. GeMoMa2 was used to examine the completeness of the coding sequences. These steps were conducted in a recursive fashion until no new candidates were detected from genome. MAFFT software (default parameters) was used for multiple alignments and then the maximum-likelihood method was used to construct the phylogenetic tree using RAxML (v 8.2.10) [75]. Furthermore, based on the results of *fos* identification, protein sequences of Fos were aligned using the Clustal W. The domain region of the Fos protein was determined using Pfam (v 1.6).

### Whole-mount in situ hybridization (WISH) analysis

To investigate the location of *fos* (ID: Mis0086400.1) gene in WT loach, a specific Dig-labelling anti-sense RNA probe was synthesized by using T7 in vitro transcriptional polymerase with DIG RNA labelling kit (Roche Molecular Biochemicals, Germany). The probe was amplified from the cDNA pool by using appropriate primers (Additional file 1: Table S5). Healthy WT loaches were selected for reproduction. Loach embryos (*n* > 60) of 72 hpf and 96 hpf were sampled. All embryos were fixed in 4% (w/v) paraformaldehyde. Then, the WISH of loach embryos was conducted according to a previously described method [77].

### Generation of fos (ID: Mis0086400.1) mutant loach

We used a CRISPR-Cas9 strategy to generate a *fos* mutant loach. The target site of CCAACTTGAGGATGAGAAATCC was determined according to all design principles. Based on our previous study, the Cas9 RNA and gRNA were transcribed in vitro. The construction methods and injection procedures were performed, referring to Sun et al. [78]. The genomic DNA was obtained from tail fin tissues by using Universal Genomic DNA Kit (CWBIO, China) according to the manufacturer’s protocol. A pair of primers that amplified the target genome region was designed for mutation analysis (Additional file 1: Table S5). The amplified DNA fragment was cloned into pMD-19T vectors and then sequenced. The *fos* mutant ones (F0 generation) were crossed with WT loach to produce F1 generation. Then, the heterozygous F1 generation with the same mutation sequences (*fos*^*+/−*^ loach) was self-crossed and the offspring was checked for homozygous mutants (*fos*^*−/−*^ loach). Quantitative PCR (qPCR) was used to detect the expression levels of *fos* in liver tissues of WT and *fos*^*−/−*^ loach. The survival rates of loach fertilized eggs were recorded. *fos*^*−/−*^ loaches were used for further analysis.

#### qPCR analysis

Total RNAs of liver tissues (*n*=9) were extracted by using RNA isoPlus (TaKaRa, Japan). qPCR conditions were as follows: 95 °C for 30 s followed by 40 cycles consisting of 95 °C for 5 s and 57 °C for 30 s. The fluorescent flux was then recorded, and the reaction continued at 72 °C for 6 s and 95 °C for 5 s. Finally, expression levels of target genes were calculated by using the 2^*−*ΔΔCT^ method. *β-actin* was used as the reference gene for normalization. All the procedures were based on the methods described by Liu et al. [79]. All of the primer sequences for qPCR are listed in Additional file 1: Table S5.

### Micro-computed tomography (Micro-CT) analysis

The whole bodies of WT, *fos*^*−/−*^ loach, and *C. sinensis* (*n*=3/group) were fixed in 4% (w/v) paraformaldehyde for 48 h. Before Micro-CT scanning, these fish were washed by phosphate buffer solution (PBS), and then scanned using Skyscan high-resolution micro-CT (CT skyscan1276, Bruker, USA). All three-dimensional (3D) images were reconstructed using the CTan1.17 and CTvox program and analyzed using DataViewer software. All fish were imaged at an isotropic voxel size of 13 μm using an X-ray tube potential of 55 kVp, a 0.50-mm aluminum filter, an X-ray intensity of 0.20 mA, and an integration time of 406 ms per slice for vertebrae. Quantitative and qualitative analyses of bone parameters were performed within a square region of interest set at 0.50 mm below the growth plate. The bone morphometric parameters including BMD, BV/TV, and Tb.Th were analyzed.

### Tartrate-resistant acid phosphatase (TRAP) staining and toluidine blue staining

The spine tissues from WT (*n*=6) and *fos*^*−/−*^ loach (*n*=6) were sampled and fixed in 4% paraformaldehyde, dehydrated using a graded alcohol series, and embedded in paraffin. Cross sections of 5-μm thickness were stained with TRAP or toluidine blue. Stained sections were observed under a light microscope (Soptop EX20, China).

### Expansion of hemoglobin genes and contraction of digestion/absorption-related genes

Genomes of the loach and other four fish species (*O. argus*, *T. tibetana*, *D. rerio*, and *C. idellus*) were used for the analysis of hemoglobin (*hb*) genes. We collected the genome sequences of *C. idellus* from NCBI (http://www.ensembl.org) for comparative analysis. To identify full complement *hb* genes in the genomes, the Hb protein sequences of zebrafish were collected according to the method described by Lei et al. [41] and used as queries to conduct TBLASTN (with *e*-value of 1×10^*−*5^) searches against each of the genomes. Gene annotations for the zebrafish *hb* gene clusters are shown in Additional file 1: Table S4. MAFFT software (default parameters) was used for multiple alignments, and then the maximum-likelihood method was used to construct the phylogenetic tree using RAxML (v 8.2.10). The contracted digestion/absorption-related genes (*atp1a1*, *atp1a2*, *atp1a3*, *ryr1*, *ryr2*, and *ryr3*) in the loach were also identified in the other four fish species genomes, and then we constructed the phylogenetic tree.

### Histological observations of posterior intestine tissues

Posterior intestine tissues were sampled from the loach (with intestinal air-breathing) and *L. elongata* (without air-breathing) (*n*=6/group), fixed in 4% paraformaldehyde for 24 h, and dehydrated in graded ethanol and embedded in paraffin wax. Cross sections of 5-μm thickness were stained with hematoxylin and eosin (H&E) and prepared for light microscopy, according to the method described by Cao and Wang [80]. Based on the results of H&E staining, we analyzed the difference of posterior intestine tissues between *M. anguillicaudatus* and *L. elongata*.

### Identification of air-breathing-related genes of the loach

To better identify air-breathing-related genes of the loach, together with our previous studies including developmental transcriptome analysis of loach posterior intestines [42], intestinal air-breathing inhibition trial (transcriptome [10] and microRNAs [43] analysis), and comparative transcriptome analysis of posterior intestines between the loach and non-air-breathing *L. elongata* [14], an air exposure trial (namely, an intestinal air-breathing enhancement trial) was performed and the procedures in details were described as Sun et al. [12]. A total of 18 WT loaches were used for transcriptome analysis. Among them, nine loaches were randomly selected for posterior intestine sampling, namely, the control group (C_chang). Then, the remaining nine individuals were placed on moist towels (air exposure group (T_chang)). After 6 h air exposure, posterior intestine tissues were sampled from T_chang groups. The samples were stored at 80 °C for RNA isolation. cDNA library of each tissue was prepared and then sequenced on the Illumina sequencing platform by Majorbio, Inc. (Shanghai, China). Gene expression levels were calculated using StringTie (v 2.1.0) with the fragments per kilobase of exon model per million mapped fragments (FPKM) method. The DEGs were identified using DESeq2. Genes with |log_2_ fold change| ≥ 1 and FDR< 0.05 were considered to be DEGs. GO functional enrichment analysis and KEGG pathway analysis were carried out using Goatools and KOBAS, respectively. Then, the RNA-Seq data was validated by qPCR. In addition, the expression levels of *hba* and *hbb* genes in posterior intestines of the loach under air exposure lasted for 12 h, were measured.

The genomes of loach and other four fish species (*O. argus*, *M. armatus*, *T. tibetana*, *D. rerio*, and *C. idellus*) were used to analyze the air-breathing-related genes. The detailed steps for the identification of the air-breathing-related genes were the same as the *fos* gene*.* Gene annotations for the air-breathing-related gene clusters in zebrafish genome are shown in Additional file 1: Table S4. These genes were identified by TBLASTN (with *e*-value of 1×10^*−*5^). MAFFT software (default parameters) was used for multiple alignments, and then the maximum-likelihood method was used to construct the phylogenetic tree using RAxML (v 8.2.10).

### Identification of FMO and UGT gene families

The genomes of the loach and other four fish species (*T. tibetana*, *D. rerio*, *O. mossambicus*, and *P. fulvidraco*) were used in the analysis of *fmo5s* and UGT gene family. We collected the genome sequences of *O. mossambicus* and *P. fulvidraco* from NCBI (http://www.ensembl.org) for comparative analysis. To identify full complement *fmo* and *ugt* genes in the genomes, the Fmo and Ugt protein sequences of zebrafish from ZFIN database (http://zfin.org/) were collected and used as queries to conduct TBLASTN (with *e*-value of 1×10^*−*5^) searches against each of the genomes. Gene annotations for the zebrafish *fmo* and *ugt* gene clusters are shown in Additional file 1: Table S4. The detailed steps for the identification of FMO and UGT gene families were the same as the *fos* gene*.* MAFFT software (default parameters) was used for multiple alignments, and then the maximum-likelihood method was used to construct the phylogenetic tree using RAxML (v 8.2.10).

### Drug stress trial

To scan the genes involved in noxious environmental adaptation of the loach, a drug stress trail (Benzopyrene (LC50=20 μg/L), 1-Naphthol (LC50=8 mg/L), Pyrene (LC50=300 μg/L), p-Nitrophenol (LC50=10 mg/L), and Bisphenol A (LC50=8 mg/L)) was performed, and WT loach adults were used here. The 24 h LC50 of different drugs were determined by our pre-experiment. After the treatment, liver tissues of nine loaches from each group (five drug treatment groups and one control group (without drug treatment)) were sampled and sequenced on the Illumina sequencing platform by Majorbio, Inc. (Shanghai, China). For each group, the liver tissues from three loaches were mixed as a biological sample. The detailed analysis steps of RNA-seq were the same as the above mentioned.

### Gene expression analysis during drug stress trail

Based on the RNA-seq results, we performed another drug stress trial. During the trial, the liver tissues (*n*=9) were sampled at different time points (0, 1, 6, 12, and 24 h) for qPCR and Western blotting (WB) analysis. All of the primer sequences for qPCR are listed in Additional file 1: Table S5. Glyceraldehyde-3-phosphate dehydrogenase (Gapdh) was used as the reference protein.

### Expression and location analysis of fmo5 (ID: Mis0185950.1) in the loach

Ten tissues (i.e., fin, blood, muscle, gill, posterior intestine, skin, spleen, barbel, liver, and anterior/mid intestine) were sampled from WT loach adults (*n*=6). Total RNAs of the tissues were extracted by using MolPure® Cell/Tissue miRNA Kit (Cat No.19331ES08; Yeasen, Shanghai, China) and used for qPCR analysis. All of the primer sequences for qPCR are listed in Additional file 1: Table S5.

The WISH analysis of loach *fmo5* was performed here. For immunohistochemical (IHC) analysis, the liver and intestine tissues from WT loach adults (*n*=6) were sampled and fixed in 4% paraformaldehyde. The procedures of immunohistochemical were performed as described previously with slight changes [81]. The polyclonal antibody of FMO5 (Rabbit, A7673, 1:1000, ABclonal, China) was used as the primary antibody and the peroxidase-conjugated goat anti-rabbit IgG (HRP Goat Anti-Rabbit IgG, ABclonal AS014, 1:10000) was used as the second antibody. Tissues were observed under a light microscope (Soptop EX20, China).

### Generation of fmo5 (ID: Mis0185950.1) mutant loach and drug stress trial

The target site of GGATGTAGAGACAGAGTCGAAGG was determined according to all design principles. The detailed steps for *fmo5* mutation generation were the same as the *fos* gene mutant*.* Moreover, qPCR and WB techniques were used to detect the expression levels of *fmo5* in liver tissues of WT (*n*=6) and *fmo5*^*−/−*^ (*n*=6) loach. The body weights of WT (*n*=30) and *fmo5*^*−/−*^ (*n*=30) loach were recorded. *fmo5*^*−/−*^ loach adults were used for further analysis.

We here conducted another drug stress trial on WT (*n*=60) and *fmo5*^*−/−*^ (*n*=60) loach, with the same 24 h LC50 of each drug. During the treatment, we recorded the survival rate every 12 h until 96 h.

### WB analysis

The liver tissues from WT and *fmo5*^*−/−*^ loach were used for WB analysis. The polyclonal antibody of FMO5 and anti-GAPDH (Rabbit, AC001, ABclonal, China) were diluted 1000 and 2000 times with the primary antibody dilution buffer (Gbcbio Technologies Inc., China), respectively. The peroxidase-conjugated goat anti-rabbit IgG (HRP Goat Anti-Rabbit IgG, ABclonal AS014, 1:10000) was selected as the second antibody. Detailed procedures were described by Ida et al. [82].

### Alcian blue-periodic acid-Schiff staining (AB-PAS staining) of loach intestines

Sections of the anterior intestine tissues from WT (*n*=6) and *fmo5*^*−/−*^ (*n*=6) loach were stained by AB-PAS staining (Nanjing Jiancheng Bioengineering Institute, Nanjing, China) according to the manufacturer’s instructions. We then measured the mucosal fold height. The number of goblet cells in each 100 μm mucosal fold height (N for short) was calculated according to the formula: *N* = (*n* (the total number of goblet cells)/*n* (the total number of mucosal folds)) / (*h* (the average height of mucosal folds)/100 μm)). The average height of mucosal folds was normalized by the length of the corresponding loach.

### Histological observations of loach liver tissues

H&E staining and immunofluorescence analysis of liver tissues from WT (*n*=6) and *fmo5*^*−/−*^ (*n*=6) loach were carried out for histological observations. The procedures of immunofluorescence were performed as described previously with slight modifications [83]. The polyclonal antibody of Chop (produced by our laboratory, Rabbit, 1:50) was used as the primary antibody and Cy3-labeled goat anti-mouse antibody (1:200, ABclonal, China) used as second antibody. The nuclei were stained with DAPI. Tissues were observed under a laser scanning confocal microscope (Leica DMi8, Germany).

### Expression analysis of ER stress-related genes in intestine and liver tissues

The intestine and liver tissues were sampled from WT (*n*=6) loach and *fmo5*^*−/−*^ (*n*=6) loach for qPCR and WB analysis. We investigated the expression profiles of some ER stress-related genes (*atf6*, *eif2ak3*, and *chop*) in liver and intestine tissues and *muc2* in intestines at mRNA and protein levels. All of the primer sequences for qPCR are listed in Additional file 1: Table S5. The polyclonal antibodies of Atf6 (Rabbit, 1:1000, A0202, ABclonal, China), Eif2ak3 (Rabbit, 1:1200, A18196, ABclonal, China), Muc2 (Rabbit, 1:1000, A14659, ABclonal, China), and Chop (produced by our laboratory) were diluted 50 times with the primary antibody dilution buffer (Gbcbio Technologies Inc., China).

### Statistical analysis

All data were presented as the mean ± standard deviation (SD). Statistical analyses were performed using SPSS 26.0 software (IBM Analytics, Richmond, VA, USA). For two group comparison, a *t*-test was performed. One-way ANOVA was performed followed by Tukey’s test for multiple comparisons. A *p* value < 0.05 was considered significant, while *p* < 0.01 and < 0.001 indicated a very significant difference and an extremely significant difference, respectively.

## Supplementary Information

**Additional file 1: Table S1.** Statistics of assembly and annotation of loach *Misgurnus anguillicaudatus* genome. (1) Statistics of Illumina HiSeq sequencing data of the loach genome. (2) 17-kmer analysis for estimation of the loach genome size. (3) Hi-C library sequencing data of the loach. (4) Statistics of repeated sequence classification of the loach genome. (5) Statistics of gene annotation of the loach genome. (6) Statistics of gene functional annotation of the loach genome. **Table S2.** Detailed gene information related to mud-dwelling behavior and intestinal evolution (air-breathing and digestion/absorption) of loach *Misgurnus anguillicaudatus*. (1) GO enrichment analysis of the expanded myosin complex genes in the loach genome. (2) KEGG enrichment analysis of the positively selected gene involved in osteoclast differentiation in the loach genome. (3) GO enrichment analysis of the expanded gene families involved in oxygen transport in the loach genome. Red and black gene IDs present *hbb* and *hba* genes, respectively. (4) KEGG enrichment analysis of the positively selected genes involved in VEGF signaling pathway in the loach genome. (5) Expression analysis of some DEGs in posterior intestine transcriptomes between *Leptobotia elongate* (LE, without air-breathing) and the loach (MA, with intestinal air-breathing) (referred from our previous study). (6) Summary of detected microRNAs and target genes involved in vascular biology of loach posterior intestines (referred from our previous study). (7) The DEGs involved in intestinal air-breathing and nutrient uptake of the loach (referred from our previous studies) (8) Expression analysis of five key DEGs in posterior intestine transcriptomes of the loach between the control (C_chang) and air exposure (T_chang) group. Un means Mis0158000.1, which is a new gene and its KEGG annotation is interleukin 1 beta (*Il1b*). (9) KEGG enrichment analysis of the contracted gene families involved in digestion/absorption of the loach. (10) KEGG enrichment analysis of the expanded genes involved in digestion/absorption of the loach. *hbb*, hemoglobin subunit beta; *hba*, hemoglobin subunit alpha; VEGF, vascular endothelial growth factor; DEGs, differentially expressed genes; *Il1b*, Interleukin-1 beta; *cldn5*, Claudin-5 Transmembrane protein deleted in VCFS; *hspb1*, heat shock protein beta-1; *vegfr1* (*flt1*), vascular endothelial growth factor receptor1; *ctgf*, Connective tissue growth factor CCN family member 2; *hif1a*, hypoxia-inducible factor 1-alpha. **Table S3.** Genes involved in detoxification function of loach *Misgurnus anguillicaudatus*. (1) The expanded genes involved in the detoxification function of the loach genome. (2) KEGG enrichment analysis of DEGs involved in the xenobiotics biodegradation and metabolism between the control and five drug stress groups. Red and black gene IDs present *fmo* and *ugt* genes, respectively. DEGs, differentially expressed genes. **Table S4.** Gene annotations for the zebrafish gene clusters in this study**.** *Fos*, proto-oncogene c-Fos; *hba*, hemoglobin subunit alpha; *hbb*, hemoglobin subunit beta; *ryr*, ryanodine receptor; *atp1a*, sodium/potassium-transporting ATPase subunit alpha; *cldn5*, claudin-5; *vegfr1*, vascular endothelial growth factor receptor 1; *hspb1*, heat shock protein beta-1; *ctgf*, connective tissue growth factor CCN family member 2; *fmo*, dimethylaniline monooxygenase [N-oxide-forming]; *ugt*, UDP-glucuronosyltransferase. **Table S5.** Primers used in this study. qPCR, quantitative PCR; # indicated T7 promoter sequences. *Fos*, proto-oncogene c-Fos; *fmo5*, dimethylaniline monooxygenase [N-oxide-forming] 5 (Mis0185930.1); *hbb*, hemoglobin subunit beta; *hba*, hemoglobin subunit alpha; *atf6*, cyclic AMP-dependent transcription factor; *eif2ak3*, eukaryotic translation initiation factor 2-alpha kinase 3; *chop*, DNA damage-inducible transcript 3 protein; *muc2*, mucin-2; Mis0185950.1, Mis0185940.1, Mis0185920.1, Mis0185970.1, Mis0186000.1, Mis0186010.1 (*fmo5*), dimethylaniline monooxygenase [N-oxide-forming] 5; Mis0115330.1, Mis0115330.1, Mis0135560.1 (*ugt2a2*), UDP-glucuronosyltransferase 2A2; Mis0072610.1 (*ugt2a1*), UDP-glucuronosyltransferase 2A1; *npr2*, atrial natriuretic peptide receptor 2; *f9*, coagulation factor IX; *hspb1*, heat shock protein beta-1; *hyou1*, hypoxia up-regulated protein 1; *ptafr*, platelet-activating factor receptor; *scx*, basic helix-loop-helix transcription factor scleraxis; *galnt8*, polypeptide N-acetylgalactosaminyltransferase 8; *krt13*, keratin, type I cytoskeletal 13 Cytokeratin-13; *gp2*, pancreatic secretory granule membrane major glycoprotein; *smco3*, single-pass membrane and coiled-coil domain-containing protein 3; *ccl5*, C-C motif chemokine 5; *ifi44*, interferon-induced protein 44; *atf4*, cyclic AMP-dependent transcription factor 4; *ugt1a1*, UDP-glucuronosyltransferase 1-1. **Table S6.** A summary of sequencing data used in genome assembly and annotation of *Misgurnus anguillicaudatus*.

**Additional file 2: Fig S1.** Genome-wide Hi-C interaction map. **Fig S2.** Divergence distributions of four TE sequences predicted by the *de novo* method (a) and comparison of the distribution of several features in the final gene set for seven fish species (b). TE, transport elements; DNA, DNA transposons; LTR, long terminal repeats; LINE, long interspersed nuclear elements; SINE, short interspersed nuclear element. The seven fish species: *Misgurnus anguillicaudatus*, *Carassius auratus*, *Cyprinus carpio*, *Danio rerio*, *Sinocyclocheilus graham*, *S. rhinocerous,* and *Triplophysa tibetana*. **Fig S3.** The construction of maximum likelihood tree of *fos* gene and generation of the *fos* (ID: Mis0086400.1) knockout loach (*fos*^*−/−*^) by CRISPR/Cas9 technology. (a) Maximum likelihood tree (TBLASTN (with *e*-value of 1×10^*−*5^)) of *fos* gene in fish species. Different branch colors represent different species. Red words indicate the knockout of *fos* gene in loach genome. **(**b) Schematic position of the CRISPR/Cas9 target site for *fos* gene knockout. (c) The transcription level of *fos* in livers of wild-type loach (WT) and *fos*^*−/−*^ loach. (d) A statistics of survival rate of fertilized eggs of WT loach and heterozygous F1 generation (F1 generation self-crossed). *** extremely significant difference (*p* < 0.001). hpf, hours post fertilization; *fos*, Proto-oncogene c-Fos. The gene in red color is a positively selected gene in the loach genome. **Fig S4.** Identification and expression analysis of air-breathing- and digestion/absorption- related genes in loach *Misgurnus anguillicaudatus*. (a) Maximum likelihood tree (TBLASTN (with *e*-value of 1×10^*−*5^)) of *hb* gene family. Different color backgrounds represent different genes. Different branch colors and inner circle colors represent different species. *hbb*, hemoglobin subunit beta; *hba*, hemoglobin subunit alpha. (b) Expression levels of *hbb* and *hba* in posterior intestines of the loach under air exposure. (c) qPCR validation of RNA-seq data of loach posterior intestines under air exposure. (d) Maximum likelihood tree (TBLASTN (with *e*-value of 1×10^*−*5^)) of air-breathing-related genes. Different branch colors represent different species. (e) Maximum likelihood tree of digestion/absorption-related genes (TBLASTN (with *e*-value of 1×10^*−*5^)). *Atp1a*, sodium/potassium-transporting ATPase subunit; *ryr*, ryanodine receptor. Different color backgrounds represent different genes. Different branch colors represent different species. *Npr2*, atrial natriuretic peptide receptor 2; *f9*, coagulation factor IX; *hspb1*, heat shock protein beta-1; *hyou1*, hypoxia up-regulated protein 1; *ptafr*, platelet-activating factor receptor; *scx*, basic helix-loop-helix transcription factor scleraxis; *galnt8*, polypeptide N-acetylgalactosaminyltransferase 8; *krt13*, keratin, type I cytoskeletal 13 Cytokeratin-13; *gp2*, pancreatic secretory granule membrane major glycoprotein; *smco3*, single-pass membrane and coiled-coil domain-containing protein 3; *ccl5*, C-C motif chemokine 5; *ifi44*, interferon-induced protein 44; *cldn5*, claudin-5 Transmembrane protein deleted in VCFS; *hspb1*, heat shock protein beta-1; *vegfr1* (*flt1*), vascular endothelial growth factor receptor 1; *ctgf*, connective tissue growth factor CCN family member 2. **Fig S5.** Identification of FMO and UGT gene families. (a) Maximum likelihood tree (TBLASTN (with *e*-value of 1×10^*−*5^)) of FMO gene family. Different colors represent different FMO gene families and different branch colors represent different species. (b) Maximum likelihood tree (TBLASTN (with *e*-value of 1×10^*−*5^)) of UGT gene family. Different colors represent different UGT gene families and different inner circle colors represent different species. **Fig S6.** qPCR validation of RNA-seq data from the drug stress trial. **Fig S7.** The expression levels of *ugt* genes in liver tissues of the loach under five drug stress. Mis115330.1 and Mis0135560.1 (*ugt2a2*), UDP-glucuronosyltransferase 2A2; Mis0072610.1 (*ugt2a1*), UDP-glucuronosyltransferase 2A1. **Fig S8.** Tissue expression analysis and the knockout of *fmo5* (ID: Mis0185930.1) gene (*fmo5*^*−*/*−*^) in loach *Misgurnus anguillicaudatus* genome. (a) Tissue expression analysis of *fmo5* (ID: Mis0185930.1) of the loach. (b) Schematic position of the CRISPR/Cas9 target site for *fmo5* gene knockout. (c) The mRNA and protein expression levels of fmo5 in livers of wild-type (WT) and *fmo5*^*−*/*−*^ loach. Different letters above error bars indicate significant difference among different tissues (*p* < 0.05); *** extremely significant difference (*p* < 0.001). **Fig S9.** Survival rates of wild-type (WT) and *fmo5*deletion (*fmo5*^*−*/*−*^) loach under five drug stress.

## Data Availability

The whole-genome assemblies of the *M. anguillicaudatus* have been submitted to NCBI under PRJNA812369 [84]. The raw reads of the RNA-seq of posterior intestine and liver tissues are submitted to NCBI under the accession number PRJNA811745 [85] and PRJNA910813 [86], respectively.

## Abbreviations

Fos: Proto-oncogene c-Fos
Hspb1: Heat shock protein beta-1
Fmo5: Flavin-containing monooxygenase 5
ABOs: Air-breathing organs
PAHs: Polycyclic aromatic hydrocarbons
LTRs: Long terminal repeats
LINEs: Long interspersed nuclear elements
CDS: Coding sequences
BUSCO: Benchmarking universal single-copy orthologues
Ma: Million years
MRCA: Most recent common ancestor
FDR: False discovery rate
PSG: Positively selected gene
hpf: Hours post-fertilization
BMD: Bone mineral density
Tb.Th: Trabecular thickness
BV/TV: Bone volume over total volume
Hb: Hemoglobin
Hif1a: Hypoxia-inducible factor 1-alpha
Vegfaa: Vascular endothelial growth factor A-A
Vegfr: Vascular endothelial growth factor receptor
Map2k2: Dual specificity mitogen-activated protein kinase kinase 2
Il1b: Interleukin 1 beta
Cldn5: Claudin-5
Ctgf: Connective tissue growth factor
UGTs: UDP-glucuronosyltransferases
BPA: Bisphenol A
DEGs: Differentially expressed genes
ER stress: Endoplasmic reticulum stress
Atf6: Cyclic AMP-dependent transcription factor
Eif2ak3: Eukaryotic translation initiation factor 2-alpha kinase 3
Chop: DNA damage-inducible transcript 3 protein
DO: Dissolved oxygen
TEs: Transport elements
WISH: Whole-mount in situ hybridization
qPCR: Quantitative PCR
Micro-CT: Micro-computed tomography
PBS: Phosphate buffer solution
TRAP: Tartrate-resistant acid phosphatase
AB-PAS staining: Alcian blue-periodic acid-Schiff staining
