# Supplementary material for: The chromosome-level genome and key genes associated with mud-dwelling behavior and adaptations of hypoxia and noxious environments in loach (Misgurnus anguillicaudatus)
Source: BMC Biol. 2023 Feb 1;21:18. doi: 10.1186/s12915-023-01517-1 (PMC9893644; doi:10.1186/s12915-023-01517-1)
Supplement: Supplementary file 1 — Additional file 1: Table S1. Statistics of assembly and annotation of loach Misgurnus anguillicaudatus genome. (1) Statistics of Illumina HiSeq sequencing data of the loach genome. (2) 17-kmer analysis for estimation of the loach genome size. (3) Hi-C library sequencing data of the loach. (4) Statistics of repeated sequence classification of the loach genome. (5) Statistics of gene annotation of the loach genome. (6) Statistics of gene functional annotation of the loach genome. Table S2. Detailed gene information related to mud-dwelling behavior and intestinal evolution (air-breathing and digestion/absorption) of loach Misgurnus anguillicaudatus. (1) GO enrichment analysis of the expanded myosin complex genes in the loach genome. (2) KEGG enrichment analysis of the positively selected gene involved in osteoclast differentiation in the loach genome. (3) GO enrichment analysis of the expanded gene families involved in oxygen transport in the loach genome. Red and black gene IDs present hbb and hba genes, respectively. (4) KEGG enrichment analysis of the positively selected genes involved in VEGF signaling pathway in the loach genome. (5) Expression analysis of some DEGs in posterior intestine transcriptomes between Leptobotia elongate (LE, without air-breathing) and the loach (MA, with intestinal air-breathing) (referred from our previous study). (6) Summary of detected microRNAs and target genes involved in vascular biology of loach posterior intestines (referred from our previous study). (7) The DEGs involved in intestinal air-breathing and nutrient uptake of the loach (referred from our previous studies) (8) Expression analysis of five key DEGs in posterior intestine transcriptomes of the loach between the control (C_chang) and air exposure (T_chang) group. Un means Mis0158000.1, which is a new gene and its KEGG annotation is interleukin 1 beta (Il1b). (9) KEGG enrichment analysis of the contracted gene families involved in digestion/absorption of the loach. (10) K [file 12915_2023_1517_MOESM1_ESM.docx]

**Additional file 1**

**Table S1. Statistics of assembly and annotation of loach *Misgurnus anguillicaudatus* genome.** (1) Statistics of Illumina HiSeq sequencing data of the loach genome. (2) 17-kmer analysis for estimation of the loach genome size. (3) Hi‐C library sequencing data of the loach. (4) Statistics of repeated sequence classification of the loach genome. (5) Statistics of gene annotation of the loach genome. (6) Statistics of gene functional annotation of the loach genome.

**Table S2. Detailed gene information related to mud-dwelling behavior and intestinal evolution (air-breathing and digestion/absorption) of loach *Misgurnus anguillicaudatus*.** (1) GO enrichment analysis of the expanded myosin complex genes in the loach genome. (2) KEGG enrichment analysis of the positively selected gene involved in osteoclast differentiation in the loach genome. (3) GO enrichment analysis of the expanded gene families involved in oxygen transport in the loach genome. Red and black gene IDs present *hbb* and *hba* genes, respectively. (4) KEGG enrichment analysis of the positively selected genes involved in VEGF signaling pathway in the loach genome. (5) Expression analysis of some DEGs in posterior intestine transcriptomes between *Leptobotia elongate* (LE, without air-breathing) and the loach (MA, with intestinal air-breathing) (referred from our previous study). (6) Summary of detected microRNAs and target genes involved in vascular biology of loach posterior intestines (referred from our previous study). (7) The DEGs involved in intestinal air-breathing and nutrient uptake of the loach (referred from our previous studies) (8) Expression analysis of five key DEGs in posterior intestine transcriptomes of the loach between the control (C_chang) and air exposure (T_chang) group. Un means Mis0158000.1, which is a new gene and its KEGG annotation is interleukin 1 beta (*Il1b*). (9) KEGG enrichment analysis of the contracted gene families involved in digestion/absorption of the loach. (10) KEGG enrichment analysis of the expanded genes involved in digestion/absorption of the loach. *hbb*, hemoglobin subunit beta; *hba*, hemoglobin subunit alpha; VEGF, vascular endothelial growth factor; DEGs, differentially expressed genes; *Il1b*, Interleukin-1 beta; *cldn5*, Claudin-5 Transmembrane protein deleted in VCFS; *hspb1*, heat shock protein beta-1; *vegfr1* (*flt1*), vascular endothelial growth factor receptor1; *ctgf*, Connective tissue growth factor CCN family member 2; *hif1a*, hypoxia-inducible factor 1-alpha.

**Table S3. Genes involved in detoxification function of loach *Misgurnus anguillicaudatus*.** (1) The expanded genes involved in the detoxification function of the loach genome. (2) KEGG enrichment analysis of DEGs involved in the xenobiotics biodegradation and metabolism between the control and five drug stress groups. Red and black gene IDs present *fmo* and *ugt* genes, respectively. DEGs, differentially expressed genes.

**Table S4. Gene annotations for the zebrafish gene clusters in this study.** *Fos*, proto-oncogene c-Fos; *hba*, hemoglobin subunit alpha; *hbb*, hemoglobin subunit beta; *ryr*, ryanodine receptor; *atp1a*, sodium/potassium-transporting ATPase subunit alpha; *cldn5*, claudin-5; *vegfr1*, vascular endothelial growth factor receptor 1; *hspb1*, heat shock protein beta-1; *ctgf*, connective tissue growth factor CCN family member 2; *fmo*, dimethylaniline monooxygenase [N-oxide-forming]; *ugt*, UDP-glucuronosyltransferase.

**Table S5. Primers used in this study.** qPCR, quantitative PCR; # indicated T7 promoter sequences. *Fos*, proto-oncogene c-Fos; *fmo5*, dimethylaniline monooxygenase [N-oxide-forming] 5 (Mis0185930.1); *hbb*, hemoglobin subunit beta; *hba*, hemoglobin subunit alpha; *atf6*, cyclic AMP-dependent transcription factor; *eif2ak3*, eukaryotic translation initiation factor 2-alpha kinase 3; *chop*, DNA damage-inducible transcript 3 protein; *muc2*, mucin-2; Mis0185950.1, Mis0185940.1, Mis0185920.1, Mis0185970.1, Mis0186000.1, Mis0186010.1 (*fmo5*), dimethylaniline monooxygenase [N-oxide-forming] 5; Mis0115330.1, Mis0115330.1, Mis0135560.1 (*ugt2a2*), UDP-glucuronosyltransferase 2A2; Mis0072610.1 (*ugt2a1*), UDP-glucuronosyltransferase 2A1; *npr2*, atrial natriuretic peptide receptor 2; *f9*, coagulation factor IX; *hspb1*, heat shock protein beta-1; *hyou1*, hypoxia up-regulated protein 1; *ptafr*, platelet-activating factor receptor; *scx*, basic helix-loop-helix transcription factor scleraxis; *galnt8*, polypeptide N-acetylgalactosaminyltransferase 8; *krt13*, keratin, type I cytoskeletal 13 Cytokeratin-13; *gp2*, pancreatic secretory granule membrane major glycoprotein; *smco3*, single-pass membrane and coiled-coil domain-containing protein 3; *ccl5*, C-C motif chemokine 5; *ifi44*, interferon-induced protein 44; *atf4*, cyclic AMP-dependent transcription factor 4; *ugt1a1*, UDP-glucuronosyltransferase 1-1.

**Table S6. A summary of sequencing data used in genome assembly and annotation of *Misgurnus anguillicaudatus*.**

**Table S1. Statistics of assembly and annotation of loach *Misgurnus anguillicaudatus* genome.**

| ^(1)^ Sample | Read number | Base count (Gb) | Clean data (Gb) | GC content (%) | | | | |
| --- | --- | --- | --- | --- | --- | --- | --- | --- |
| loach | 990,839,450 | 148.62 | 148 | 39 | | | | |
| ^(2)^ Sample | *K*-mer number | *K*-mer depth | Genome  size (Mb) | Revised genome  size (Mb) | Repeat  (%) | Heterozygous  rate (%) | | |
| loach | 109,334,065,638 | 90 | 1,150 | 1,135 | 57.60 | 1.49 | | |
| ^(3)^ Sample | Raw reads number | Raw bases  (bp) | Clean reads number | Clean bases  (bp) | Read length  (bp) | GC content (%) | | Chromosome mounting rate (%) |
| loach | 569,812,836 | 85,471,925,400 | 562,124,346 | 80,009,423,782 | 150 | 36.36 | | 98.66 |
| ^(4)^ | RepBase TEs | | TE proteins (bp) | | De novo | | Combined TEs | |
|  | Length (bp) | % in genome | Length (bp) | % in genome | Length (bp) | % in genome | Length (bp) | % in genome |
| DNA | 229,590,838 | 19.56 | 16,422,566 | 1.40 | 462,372,211 | 39.39 | 569,872,585 | 48.54 |
| LINE | 28,958,062 | 2.47 | 20,381,428 | 1.74 | 82,451,266 | 7.02 | 97,255,715 | 8.28 |
| SINE | 3,911,545 | 0.33 | 0 | 0.00 | 7,149,191 | 0.61 | 10,601,438 | 0.90 |
| LTR | 42,527,475 | 3.62 | 28,874,932 | 2.46 | 167,004,418 | 14.23 | 180,053,077 | 15.34 |
| Satellite | 5,859,186 | 0.50 | 0 | 0.00 | 13,052,375 | 1.11 | 18,734,646 | 1.60 |
| Simple repeat | 0 | 0.00 | 0 | 0.00 | 36,435 | 0.00 | 36,435 | 0.00 |
| Other | 4,676 | 0.00 | 177 | 0.00 | 0 | 0.00 | 4,853 | 0.00 |
| Unknown | 2,624,975 | 0.22 | 6,087 | 0.00 | 23,931,876 | 2.04 | 26,525,210 | 2.26 |
| Total | 305,675,507 | 26.04 | 65,670,591 | 5.59 | 673,218,169 | 57.35 | 785,575,487 | 66.92 |
| ^(5)^ Gene set | Protein coding gene number | Average gene length (bp) | Average CDS length (bp) | Average exon per gene | Average exon length (bp) | Average intron length (bp) | | |
| denovo/Genscan | 24,371 | 30,372.39 | 1,590.36 | 8.41 | 189.19 | 3,886.24 | | |
| denovo/AUGUSTUS | 25,071 | 16,573.76 | 1,389.93 | 8.11 | 171.48 | 2,136.95 | | |
| homo/*Danio rerio* | 136,172 | 11,164.8 | 1,023.12 | 3.13 | 326.48 | 4,752.95 | | |
| homo/*Carassius auratus* | 85,200 | 11,301.63 | 1,134.89 | 3.84 | 295.58 | 3,580.38 | | |
| homo/*Cyprinus carpio* | 84,333 | 6,358.18 | 876.88 | 3.33 | 263.28 | 2,351.83 | | |
| homo/*Sinocyclocheilus grahami* | 84,675 | 9,206.75 | 800.29 | 3.65 | 219.22 | 3,171.52 | | |
| homo/*Sinocyclocheilus rhinocerous* | 74,984 | 10,595.4 | 932.16 | 3.96 | 235.45 | 3,265.69 | | |
| trans.orf/RNAseq | 17,434 | 25,818.97 | 1,826.42 | 11.22 | 282.05 | 2,217.27 | | |
| BUSCO | 4,903 | 18,575.28 | 1,965.42 | 13.2 | 148.91 | 1,361.59 | | |
| MAKER | 25,686 | 25,031.78 | 1,563.09 | 9.2 | 251.41 | 2,769.38 | | |
| HiCESAP | 24,974 | 22,413.7 | 1,678.34 | 9.96 | 259.43 | 2,213.89 | | |
| ^(6)^ |  | | Number | Percent (%) | | | | |
| Total |  | | 24,974 |  | | | | |
|  | Annotated |  | 24,298 | 97.29 | | | | |
|  |  | InterPro | 22,259 | 89.13 | | | | |
|  |  | GO | 16,935 | 67.81 | | | | |
|  |  | KEGG_ALL | 23,645 | 94.68 | | | | |
|  |  | KEGG_KO | 14,255 | 57.08 | | | | |
|  |  | Swissprot | 20,755 | 83.11 | | | | |
|  |  | TrEMBL | 23,770 | 95.18 | | | | |
|  |  | TF | 3,792 | 15.18 | | | | |
|  |  | Pfam | 21,413 | 85.74 | | | | |
|  |  | NR | 24,051 | 96.30 | | | | |
|  |  | KOG | 19,261 | 77.12 | | | | |
|  | Unannotated |  | 676 | 2.71 | | | | |

(1) Statistics of Illumina HiSeq sequencing data of the loach genome. (2) 17-kmer analysis for estimation of the loach genome size. (3) Hi‐C library sequencing data of the loach. (4) Statistics of repeated sequence classification of the loach genome. (5) Statistics of gene annotation of the loach genome. (6) Statistics of gene functional annotation of the loach genome.

**Table S2. Detailed gene information related to mud-dwelling behavior and intestinal evolution (air-breathing and digestion/absorption) of loach *Misgurnus anguillicaudatus*.**

| ^(1)^ GO terms | GO ID | P-value | Gene ID | | |
| --- | --- | --- | --- | --- | --- |
| Myosin complex | GO:0016459 | 1.55E-16 | Mis0182300.1, Mis0227920.1, Mis0172410.1, Mis0134630.1, Mis0149810.1, Mis0229500.1,  Mis0205460.1, Mis0235290.1, Mis0153580.1, Mis0019240.1, Mis0205270.1, Mis0055220.1,  Mis0153570.1, Mis0231000.1, Mis0085330.1, Mis0201130.1, Mis0229450.1, Mis0205500.1,  Mis0102110.1, Mis0235990.1, Mis0231050.1, Mis0134640.1, Mis0055210.1, Mis0154970.1 | | |
| ^(2)^ KEGG pathway | Pathway ID | P-value | Gene ID (name) | | |
| Osteoclast differentiation | ko04380 | 0.167879514 | Mis0086400.1 (*fos*, proto-oncogene c-Fos) | | |
| ^(3)^ GO term | GO ID | P-value | Gene id | | |
| Oxygen binding | GO:0019825 | 3.11E-12 | Mis0119320.1, Mis0119420.1, Mis0119410.1, Mis0119380.1, Mis0119400.1, Mis0119430.1, Mis0119310.1, Mis0119390.1, Mis0119330.1, Mis0119370.1, Mis0119350.1, Mis0119340.1 | | |
| Gas transport | GO:0015669 | 1.48E-12 |  |  |  |
| Oxygen transport | GO:0015671 | 1.48E-12 |  |  |  |
| Hemoglobin complex | GO:0005833 | 2.86E-13 |  |  |  |
| ^(4)^ KEGG pathway | Pathway ID | P-value | Gene ID | Gene name | |
| VEGF signaling pathway | ko04370 | 9.88E-01 | Mis0213370.1 | *hspb1* or *hsp27* (heat shock protein beta-1) | |
| ^(5)^ Seq id | Gene name | LE | MA | log_2_ (MA/LE) | P value (MA/LE) |
| ORTHOMCL12415 | Un (*il1b*) | 18.85 | 40.64 | 1.1 | 1.09E-03 |
| ORTHOMCL17173 | *cldn5* | 33.3 | 156.43 | 2.23 | 1.14E-04 |
| ORTHOMCL15176 | *hspb1* | 7.42 | 9.28 | 0.32 | 1.28E-03 |
| ORTHOMCL18201 | *vegfr1* | 2.93 | 8.83 | 1.56 | 1.28E-03 |
| ORTHOMCL10309 | *ctgf* | 53.7 | 135.14 | 1.33 | 5.38E-11 |
| ^(6)^ MicroRNAs | Target genes | | | | |
| miR-15/107 cluster | *hif1a*, *vegf* | | | | |
| miR-17/92 cluster | *ctgf* | | | | |
| miR-200 family | *vegf* and *vegfr1* | | | | |
| ^(7)^ Gene ID | Gene name | | | | |
| *vegfr1* | vascular endothelial growth factor receptor1 | | | | |
| *egfr* | epidermal growth factor receptor | | | | |
| *vegfaa* | vascular endothelial growth factor AA | | | | |
| ^(8)^ Gene ID | Gene name | C_chang FPKM | T_chang FPKM | log_2_ (T_chang/C_chang) | P value (C_chang-vs-T_chang) |
| Mis0158000.1 | Un (il1b) | 4.143333333 | 21.35 | 2.410431839 | 1.82E-07 |
| Mis0104630.1 | *cldn5* | 185.0166667 | 468.87 | 1.388317798 | 6.08E-05 |
| Mis0213370.1 | *hspb1* | 327.1466667 | 805.29 | 1.260889944 | 0.002462536 |
| Mis0005600.1 | *vegfr1* (*flt1*) | 10.55666667 | 20.64333333 | 1.029326743 | 0.000235274 |
| Mis0028860.1 | *ctgf* | 102.4166667 | 322.43 | 1.688438639 | 0.000250328 |
| ^(9)^ Pathway Hierarchy | KEGG pathway | Pathway ID | P-value | Gene ID | |
| Digestion/absorption | Salivary secretion | ko04970 | 6.50E-10 | Mis0192220.1, Mis0092600.1, Mis0099610.1, Mis0092610.1, Mis0024720.1,  Mis0027830.1, Mis0092590.1, Mis0015860.1, Mis0125530.1 | |
|  | Mineral absorption | ko04978 | 2.17E-09 | Mis0192220.1, Mis0092600.1, Mis0092610.1, Mis0024720.1,  Mis0092590.1, Mis0015860.1, Mis0125530.1 | |
|  | Carbohydrate digestion and absorption | ko04973 | 3.96E-09 | Mis0192220.1, Mis0092600.1, Mis0092610.1, Mis0024720.1,  Mis0092590.1, Mis0015860.1, Mis0125530.1 | |
|  | Pancreatic secretion | ko04972 | 1.05E-07 | Mis0192220.1, Mis0092600.1, Mis0092610.1, Mis0024720.1, Mis0097270.1,  Mis0199350.1, Mis0092590.1, Mis0015860.1, Mis0125530.1 | |
|  | Protein digestion and absorption | ko04974 | 2.67E-07 | Mis0192220.1, Mis0092600.1, Mis0092610.1, Mis0024720.1, Mis0092590.1,  Mis0125530.1, Mis0015860.1 | |
|  | Bile secretion | ko04976 | 1.52E-06 | Mis0192220.1, Mis0092600.1, Mis0092610.1, Mis0024720.1, Mis0092590.1,  Mis0015860.1, Mis0125530.1 | |
|  | Gastric acid secretion | ko04971 | 2.32E-06 | Mis0192220.1, Mis0092600.1, Mis0092610.1, Mis0024720.1, Mis0092590.1,  Mis0015860.1, Mis0125530.1 | |
| ^(10)^ Pathway hierarchy | KEGG pathway | Pathway ID | P-value | Gene ID | |
| Digestion/absorption | Pancreatic secretion | ko04972 | 5.50E-01 | Mis0219230.1, Mis0019870.1 | |

(1) GO enrichment analysis of the expanded myosin complex genes in the loach genome. (2) KEGG enrichment analysis of the positively selected gene involved in osteoclast differentiation in the loach genome. (3) GO enrichment analysis of the expanded gene families involved in oxygen transport in the loach genome. Red and black gene IDs present *hbb* and *hba* genes, respectively. (4) KEGG enrichment analysis of the positively selected genes involved in VEGF signaling pathway in the loach genome. (5) Expression analysis of some DEGs in posterior intestine transcriptomes between *Leptobotia elongate* (LE, without air-breathing) and the loach (MA, with intestinal air-breathing) (referred from our previous study). (6) Summary of detected microRNAs and target genes involved in vascular biology of loach posterior intestines (referred from our previous study). (7) The DEGs involved in intestinal air-breathing and nutrient uptake of the loach (referred from our previous studies) (8) Expression analysis of five key DEGs in posterior intestine transcriptomes of the loach between the control (C_chang) and air exposure (T_chang) group. Un means Mis0158000.1, which is a new gene and its KEGG annotation is interleukin 1 beta (*Il1b*). (9) KEGG enrichment analysis of the contracted gene families involved in digestion/absorption of the loach. (10) KEGG enrichment analysis of the expanded genes involved in digestion/absorption of the loach. *hbb*, hemoglobin subunit beta; *hba*, hemoglobin subunit alpha; VEGF, vascular endothelial growth factor; DEGs, differentially expressed genes; *Il1b*, Interleukin-1 beta; *cldn5*, Claudin-5 Transmembrane protein deleted in VCFS; *hspb1*, heat shock protein beta-1; *vegfr1* (*flt1*), vascular endothelial growth factor receptor1; *ctgf*, Connective tissue growth factor CCN family member 2; *hif1a*, hypoxia-inducible factor 1-alpha.

**Table S3. Genes involved in detoxification function of loach *Misgurnus anguillicaudatus*.**

| ^(1)^ Pathway hierarchy | KEGG pathway | Pathway ID | P-value | Gene ID |
| --- | --- | --- | --- | --- |
| Xenobiotics biodegradation and metabolism | Metabolism of xenobiotics by cytochrome P450 | ko00983 | 1.06E-05 | Mis0072610.1, Mis0120920.1, Mis0182860.1, Mis0135560.1, Mis0182880.1 |
|  | Drug metabolism-other enzymes | ko00980 | 2.55E-05 | Mis0072610.1, Mis0120920.1, Mis0182860.1, Mis0135560.1, Mis0182880.1 |
|  | Drug metabolism-cytochrome P450 | ko00982 | 3.60E-18 | Mis0072610.1, Mis0185970.1, Mis0186000.1, Mis0185950.1, Mis0185960.1, Mis0135560.1, Mis0186020.1, Mis0185930.1, Mis0185920.1, Mis0120920.1, Mis0182860.1, Mis0185940.1, Mis0182880.1, Mis0186010.1 |
| ^(2)^ Drugs | KEGG pathway | Pathway ID | P-value | Gene ID |
| Benzopyrene | Xenobiotics biodegradation and metabolism | Map00982 | 0.015551211 | Mis0185970.1, Mis0185930.1, Mis0185950.1, Mis0138270.1, Mis0236610.1, Mis0070270.1, Mis0115360.1, Mis0138300.1, Mis0115340.1, Mis0182880.1, Mis0072610.1, Mis0115350.1, Mis0138290.1 |
| 1-Naphthol |  |  | 2.62E-08 | Mis0185970.1, Mis0185930.1, Mis0185950.1, Mis182860.1, Mis186010.1, Mis0046720.1, Mis0138270.1, Mis0236610.1, Mis0070270.1, Mis0115360.1, Mis0138300.1, Mis0115340.1, Mis0182880.1, Mis0072610.1, Mis0115350.1, Mis0138290.1, Mis0047710.1, Mis0120920.1 |
| Pyrene |  |  | 0.001922853 | Mis0185970.1, Mis0185930.1, Mis0185950.1, Mis0186000.1, Mis0070270.1, Mis0046720.1, Mis0138300.1, Mis0072610.1, Mis0138290.1 |
| p-Nitrophenol |  |  | 0.000224248 | Mis0185970.1, Mis0185930.1, Mis0185950.1, Mis0138270.1, Mis0236610.1, Mis0070270.1, Mis0115360.1, Mis0138300.1, Mis0115340.1, Mis0182880.1, Mis0072610.1, Mis0115350.1, Mis0138290.1 |
| Bisphenol A |  |  | 3.43E-08 | Mis0185970.1, Mis0185930.1, Mis0185950.1, Mis0186000.1, Mis0186010.1, Mis182860.1, Mis186010.1, Mis0046720.1, Mis0236610.1, Mis0070270.1, Mis0115360.1, Mis0138300.1, Mis0115340.1, Mis0182880.1, Mis0072610.1, Mis0115350.1, Mis0138290.1, Mis0120920.1 |

(1) The expanded genes involved in the detoxification function of the loach genome. (2) KEGG enrichment analysis of DEGs involved in the xenobiotics biodegradation and metabolism between the control and five drug stress group. Red and black gene IDs present *fmo* and *ugt* genes, respectively. DEGs, differentially expressed genes.

**Table S4. Gene annotations for the zebrafish gene clusters in this study.**

| Gene name | ID |
| --- | --- |
| Zebrafish *fos* | DRER-rna46673, DRER-rna52499, DRER-rna48067 |
| Zebrafish *hba* | ENSDARG00000089124, ENSDARG00000088330, ENSDARG00000089475, ENSDARG00000045142, ENSDARG00000069735, ENSDARG00000097011, ENSDARG00000079078, ENSDARG00000045144 |
| Zebrafish *hbb* | ENSDARG00000010918, ENSDARG00000017367, ENSDARG00000069734, ENSDARG00000097238, ENSDARG00000089087, ENSDARG00000038147, ENSDARG00000113599, ENSDARG00000115405, ENSDARG00000087390 |
| Zebrafish *ryr* | ZDB-GENE-061226-3, ZDB-GENE-070705-417, ZDB-GENE-041001-165, ZDB-GENE-020108-2, ZDB-GENE-071001-1 |
| Zebrafish *atp1a* | ZDB-GENE-001212-5, ZDB-GENE-001212-6, ZDB-GENE-001212-8, ZDB-GENE-001212-7, ZDB-GENE-001212-1, ZDB-GENE-001212-4, ZDB-GENE-001212-2, ZDB-GENE-020501-1, ZDB-GENE-001212-3 |
| Zebrafish *cldn5* | ZDB-GENE-040426-2442, ZDB-GENE-041010-140 |
| Zebrafish *vegfr1* | ZDB-GENE-050407-1 |
| Zebrafish *hspb1* | ZDB-GENE-030326-4 |
| Zebrafish *ctgf* | ZDB-GENE-070705-82, ZDB-GENE-030131-102 |
| Zebrafish *fmo* | ZDB-GENE-030131-6606, ZDB-GENE-041001-98, ZDB-GENE-031010-24, 793236, ZDB-GENE-030131-2644 |
| Zebrafish *ugt* | ZDB-GENE-080227-7, ZDB-GENE-080227-6, ZDB-GENE-040426-2762, ZDB-GENE-071004-4, ZDB-GENE-071004-5, ZDB-GENE-080227-3, ZDB-GENE-080227-5, ZDB-GENE-080227-11, ZDB-GENE-080227-12, ZDB-GENE-080227-14, ZDB-GENE-080227-13, ZDB-GENE-080227-15, ZDB-GENE-080227-10, ZDB-GENE-100402-2, ZDB-GENE-100402-1, ZDB-GENE-100402-3, ZDB-GENE-100402-4, ZDB-GENE-060929-796, ZDB-GENE-081104-3, ZDB-GENE-080721-22, ZDB-GENE-080721-21, ZDB-GENE-080721-23, ZDB-GENE-080721-20, ZDB-GENE-080305-10, ZDB-GENE-081028-66, ZDB-GENE-091118-36, ZDB-GENE-060825-206, ZDB-GENE-050419-68, ZDB-GENE-050419-23, ZDB-GENE-100406-6, ZDB-GENE-160628-4, ZDB-GENE-100406-3, ZDB-GENE-100406-4, ZDB-GENE-100406-5, ZDB-GENE-051120-60, ZDB-GENE-100406-2, ZDB-GENE-100406-1, ZDB-GENE-061103-373, ZDB-GENE-030131-1097, ZDB-GENE-081105-106 |

*Fos*, proto-oncogene c-Fos; *hba*, hemoglobin subunit alpha; *hbb*, hemoglobin subunit beta; *ryr*, ryanodine receptor; *atp1a*, sodium/potassium-transporting ATPase subunit alpha; *cldn5*, claudin-5; *vegfr1*, vascular endothelial growth factor receptor 1; *hspb1*, heat shock protein beta-1; *ctgf*, connective tissue growth factor CCN family member 2; *fmo*, dimethylaniline monooxygenase [N-oxide-forming]; *ugt*, UDP-glucuronosyltransferase.

**Table S5. Primers used in this study.**

| Gene names | Primer sequences (5ʹ- 3ʹ) | Gene names | Primer sequences (5ʹ- 3ʹ) |  |
| --- | --- | --- | --- | --- |
| Primers for whole-mount *in situ* hybridization analysis | | Primers for qPCR | |  |
| *fos*-F | GACATTGCCAACCTGCTCAA |  |  |  |
| *fos*-R | GTGTGAATCTCAGGGACCGA | Mis0185950.1-q-F | GAGACACATGAGGCAGAACG |  |
| *fos*-T7-R | (TAATACGACTCACTATAGG)**^#^** GTGTGAATCTCAGGGACCGA | Mis0185950.1-q-R | AGAAATCCGGCCTCTGTCTC |  |
| *fmo5*-F | GAGGCAATGGCTCGAAGGTA | Mis0185940.1-q-F | GATTATCCCATCCCTGCCCA |  |
| *fmo5*-R | TTAGCGAGACAGACGAGTGC | Mis0185940.1-q-R | AGAGTGAGGAAAGTC TGGCC |  |
| *fmo5*-T7-R | (TAATACGACTCACTATAGG)**^#^**  TTAGCGAGACAGACGAGTGC | Mis0185920.1-q-F  Mis0185920.1-q-R | CAATGGAAAGGTGCACGACA  AGCGTGTACAGACTTTCCCT |  |
| Primers for mutation analysis | | Mis0185970.1-q-F | GGACAGAGATGGACGAGAGG |  |
| *fos*-mu-F | AAACAAGATGGCAGCAGCAAAA | Mis0185970.1-q-R | TGCGGGTACTCAGAAACACT |  |
| *fos*-mu-R | AGATCCTCACCGTGGAGTTG | Mis0186000.1-q-F | GAGACACATGAGGCAGAACG |  |
| *fmo5*-mu-F | CCATTCCTGCCCACTTTCCA | Mis0186000.1-q-R CCTCTCGTCCATCTCTGTCC | |  |
| *fmo5*-mu-R AGACCCCTGGCCTTCGATAA | | *npr2*-F | TACCGCATCGCATATAGC |  |
| Primers for qPCR | | *npr2*-R  *f9*-F | ACAGCCAAGAACTTCCATT  GTGACTGTGCTGATGGTTA |  |
| *β-actin*-q-F | TTCCTGGGTATGGAGTCTTGCG |  |  |  |
| *β-actin*-q-R | AGAGGTTTAGGTTGGTCGTTTG | *f9*-R | TCCTTCGTTCTTCGTGATG |  |
| *fos*-q-F | CGGTCCCTGAGATTCACACC | *hspb1*-F | AGATGGAGTGGTGGAGATTA |  |
| *fos*-q-R CATCCGCAGACACAAACGTC | | *hspb1*-R | AGATGTGCT GACTGTGTTC |  |
| *hbb*-q-F | ACCACTCTTCAGGACATCT | *hyou1*-F | TGCAAGACCTTACTGACCGT |  |
| *hbb*-q-R | ACCAACAGCGAGGAACTTC | *hyou1*-R | GCTCTTTAGTGTTGGCCGAG |  |
| *hba*-q-F | ACAAGTCTGTCGTGAAGGCC | *ptafr*-F | AAGCAATACACCACAGGAT |  |
| *hba*-q-R | CAATCTTTGACACGGCCTCA | *ptafr* -R | CATAACGAGACTGATGATACG |  |
| Mis0186010.1-q-F | GCCTTCTGCCTCAAAGTCTG | *scx*-F | ACTTCCTGTGCTCTGAGA | |
| Mis0186010.1-q-R | ATCTTCTCTTCAGCCGTCCC | *scx*-R | TGGTGGTTGTGTTGATGAT | |
| Mis0115330.1-q-F | CATGGGGTGCCTATTTTGGG | *glant8*-F | GTTGTTCAGTATCAGCCATC | |
| Mis0115330.1-q-R | ACTCAATCCAGAAGACGGCA | *glant8*-R | AATCTCCATTCCTTCATCCA | |
| Mis0072610.1-q-F | CAACCCCACGGAAACAGAAG | *krt13*-F | GGCTGAACTTACTGTGAATG | |
| Mis0072610.1-q-R | GCAGCAGGTCAAAGTTAGCA | *krt13*-R | TCAACAACACTCGCATCC | |
| Mis0135560.1-q-F | TGATGTTGACTGATCCGGCT | *gp2*-F | CAGTGAGGTTGAGGATGATGAG | |
| Mis0135560.1-q-R | GAAAGGTCATGCGATCCGTC | *gp2*-R | GTGGTGTAGTTAGTTGCTCTCC | |
| *fmo5*-q-F | GAGGCAGAGCGTATTTGTGG | *smco3*-F | TGTGCTTCAATCCAATTACC | |
| *fmo5*-q-R | CAAACACCACACATGAGCCA | *smco3*-R | TGCTTGCCAGAATCGTTA | |
| *atf6*-q-F | CAACCTCTCCAGACCACAGT | *ccl5*-F | GGTAGTCACTTGGCTCCAGT | |
| *atf6*-q-R | AACCACAGGAGACGCTGTTA | *ccl5*-R | TTGCATGGCTCTTTTCACCC | |
| *eif2ak3*-q-F | GTCCGCAAGTTCTTCCATCC | *ifi44*-F | CGTGAAGGATGGCTATGAA | |
| *eif2ak3*-q-R | CCACTTTGTTCCGTGCTTCA | *ifi44-*R | GGCTACTATCAATCAGATGGT | |
| *chop*-q-F | TCCGCCTTCAGACGCTCGTT | *atf4*-F | TGTCGTCGCTCTCGTGGCTT | |
| *chop*-q-R  *muc2*-q-F | CCTCTCCACAACCACCATCT  TAGATGCCTCACCACACGTT | *atf4*-R  *ugt1a1*-F | TGTCGTCGCTCTCGTGGCTT  TCGAGTACCCCAGACCCTTA | |
| *muc2*-q-R | GTCCGCAAGTTCTTCCATCC | *ugt1a1*-R | CCTCCATAGCACCCTCTGAG | |
| Mis0085380.1-q-F | CACATGGTGGCTGGATATGC | Mis0186010.1-q-F | GCCTTCTGCCTCAAAGTCTG | |
| Mis0085380.1-q-R | CGCATGTGATGACCTTCGAG | Mis0186010.1-q-R | ATCTTCTCTTCAGCCGTCCC | |
| Mis0151600.1-q-F | GGAGAACATCAATGGCGGTC | Mis0046720.1-q-F | AGGAAGATGGCGTTCGAGAA | |
| Mis0151600.1-q-R | CCACGATGCGAAAGGTGTAG | Mis0046720.1-q-R | TGAGAGATGATGCTGCCACA | |
| Mis0057070.1-q-F | GATTCTGCCCTTTTGCCCAA | Mis0190440.1-q-F | GGACAGACAATGGGAGGACA | |
| Mis0057070.1-q-R | ACAGGTGATTGGTGACTCGT | Mis0190440.1-q-R | ACCAAACAATGAGCGTGTCC | |

qPCR, quantitative PCR; # indicated T7 promoter sequences. *Fos*, proto-oncogene c-Fos; *fmo5*, dimethylaniline monooxygenase [N-oxide-forming] 5 (Mis0185930.1); *hbb*, hemoglobin subunit beta; *hba*, hemoglobin subunit alpha; *atf6*, cyclic AMP-dependent transcription factor; *eif2ak3*, eukaryotic translation initiation factor 2-alpha kinase 3; *chop*, DNA damage-inducible transcript 3 protein; *muc2*, mucin-2; Mis0185950.1, Mis0185940.1, Mis0185920.1, Mis0185970.1, Mis0186000.1, Mis0186010.1 (*fmo5*), dimethylaniline monooxygenase [N-oxide-forming] 5; Mis0115330.1, Mis0115330.1, Mis0135560.1 (*ugt2a2*), UDP-glucuronosyltransferase 2A2; Mis0072610.1 (*ugt2a1*), UDP-glucuronosyltransferase 2A1; *npr2*, atrial natriuretic peptide receptor 2; *f9*, coagulation factor IX; *hspb1*, heat shock protein beta-1; *hyou1*, hypoxia up-regulated protein 1; *ptafr*, platelet-activating factor receptor; *scx*, basic helix-loop-helix transcription factor scleraxis; *galnt8*, polypeptide N-acetylgalactosaminyltransferase 8; *krt13*, keratin, type I cytoskeletal 13 Cytokeratin-13; *gp2*, pancreatic secretory granule membrane major glycoprotein; *smco3*, single-pass membrane and coiled-coil domain-containing protein 3; *ccl5*, C-C motif chemokine 5; *ifi44*, interferon-induced protein 44; *atf4*, cyclic AMP-dependent transcription factor 4; *ugt1a1*, UDP-glucuronosyltransferase 1-1; Mis0085380.1 (*cdc40*), pre-mRNA-processing factor 17 Cell division cycle 40 homolog PRP17 homolog; Mis0151600.1 (*epha8*), ephrin type-A receptor 8; Mis0046720.1 (*ugt1a1*), UDP-glucuronosyltransferase 1-1; Mis0057070.1 (*gsto1*), glutathione S-transferase omega-1; Mis0190440.1 (*ppp4r3b*), serine/threonine-protein phosphatase 4 regulatory subunit 3.

**Table S6. A summary of sequencing data used in genome assembly and annotation of *Misgurnus anguillicaudatus*.**

| Library types | Insert size（bp） | Raw data (Gb) | Clean data (Gb) | Read length (bp) |
| --- | --- | --- | --- | --- |
| Illumina reads | 350 | 145.11 | 148.63 | 150 |
| PacBio reads | 15000 | 185 | / | 13390 |
| Hi-C reads | 100-500 | 85.47 | 80 | 150 |
| RNA reads | 350 | 13.06 | 13.23 | 150 |
