# Supplementary material for: The chromosome-level genome and key genes associated with mud-dwelling behavior and adaptations of hypoxia and noxious environments in loach (Misgurnus anguillicaudatus)
Source: BMC Biol. 2023 Feb 1;21:18. doi: 10.1186/s12915-023-01517-1 (PMC9893644; doi:10.1186/s12915-023-01517-1)
Supplement: Supplementary file 2 — Additional file 2: Fig S1. Genome-wide Hi-C interaction map. Fig S2. Divergence distributions of four TE sequences predicted by the de novo method (a) and comparison of the distribution of several features in the final gene set for seven fish species (b). TE, transport elements; DNA, DNA transposons; LTR, long terminal repeats; LINE, long interspersed nuclear elements; SINE, short interspersed nuclear element. The seven fish species: Misgurnus anguillicaudatus, Carassius auratus, Cyprinus carpio, Danio rerio, Sinocyclocheilus graham, S. rhinocerous, and Triplophysa tibetana. Fig S3. The construction of maximum likelihood tree of fos gene and generation of the fos (ID: Mis0086400.1) knockout loach (fos−/−) by CRISPR/Cas9 technology. (a) Maximum likelihood tree (TBLASTN (with e-value of 1×10^−5^)) of fos gene in fish species. Different branch colors represent different species. Red words indicate the knockout of fos gene in loach genome. (b) Schematic position of the CRISPR/Cas9 target site for fos gene knockout. (c) The transcription level of fos in livers of wild-type loach (WT) and fos−/− loach. (d) A statistics of survival rate of fertilized eggs of WT loach and heterozygous F1 generation (F1 generation self-crossed). *** extremely significant difference (p < 0.001). hpf, hours post fertilization; fos, Proto-oncogene c-Fos. The gene in red color is a positively selected gene in the loach genome. Fig S4. Identification and expression analysis of air-breathing- and digestion/absorption- related genes in loach Misgurnus anguillicaudatus. (a) Maximum likelihood tree (TBLASTN (with e-value of 1×10^−5^)) of hb gene family. Different color backgrounds represent different genes. Different branch colors and inner circle colors represent different species. hbb, hemoglobin subunit beta; hba, hemoglobin subunit alpha. (b) Expression levels of hbb and hba in posterior intestines of the loach under air exposure. (c) qPCR validation of RNA-seq data of loach posterior intestines unde [file 12915_2023_1517_MOESM2_ESM.docx]

**Additional file 2**

**Figure S1. Genome-wide Hi-C interaction map.**

**Figure S2. Divergence distributions of four TE sequences predicted by the *de novo* method (a) and comparison of the distribution of several features in the final gene set for seven fish species (b).** TE, transport elements; DNA, DNA transposons; LTR, long terminal repeats; LINE, long interspersed nuclear elements; SINE, short interspersed nuclear element. The seven fish species: *Misgurnus anguillicaudatus*, *Carassius auratus*, *Cyprinus carpio*, *Danio rerio*, *Sinocyclocheilus graham*, *S. rhinocerous,* and *Triplophysa tibetana*.

**Figure S3. The construction of maximum likelihood tree of *fos* gene and generation of the *fos* (ID: Mis0086400.1) knockout loach (*fos^-/-^*) by CRISPR/Cas9 technology.** (a) Maximum likelihood tree (TBLASTN (with *e*-value of 1×10^-5^)) of *fos* gene in fish species. Different branch colors represent different species. Red words indicate the knockout of *fos* gene in loach genome. **(**b) Schematic position of the CRISPR/Cas9 target site for *fos* gene knockout. (c) The transcription level of *fos* in livers of wild-type loach (WT) and *fos^-/-^* loach. (d) A statistics of survival rate of fertilized eggs of WT loach and heterozygous F1 generation (F1 generation self-crossed). *** extremely significant difference (*p* < 0.001). hpf, hours post fertilization; *fos*, Proto-oncogene c-Fos. The gene in red color is a positively selected gene in the loach genome.

**Figure S4. Identification and expression analysis of air-breathing- and digestion/absorption- related genes in loach *Misgurnus anguillicaudatus*.** (a) Maximum likelihood tree (TBLASTN (with *e*-value of 1×10^-5^)) of *hb* gene family. Different color backgrounds represent different genes. Different branch colors and inner circle colors represent different species. *hbb*, hemoglobin subunit beta; *hba*, hemoglobin subunit alpha. (b) Expression levels of *hbb* and *hba* in posterior intestines of the loach under air exposure. (c) qPCR validation of RNA-seq data of loach posterior intestines under air exposure. (d) Maximum likelihood tree (TBLASTN (with *e*-value of 1×10^-5^)) of air-breathing-related genes. Different branch colors represent different species. (e) Maximum likelihood tree of digestion/absorption-related genes (TBLASTN (with *e*-value of 1×10^-5^)). *Atp1a*, sodium/potassium-transporting ATPase subunit; *ryr*, ryanodine receptor. Different color backgrounds represent different genes. Different branch colors represent different species. *Npr2*, atrial natriuretic peptide receptor 2; *f9*, coagulation factor IX; *hspb1*, heat shock protein beta-1; *hyou1*, hypoxia up-regulated protein 1; *ptafr*, platelet-activating factor receptor; *scx*, basic helix-loop-helix transcription factor scleraxis; *galnt8*, polypeptide N-acetylgalactosaminyltransferase 8; *krt13*, keratin, type I cytoskeletal 13 Cytokeratin-13; *gp2*, pancreatic secretory granule membrane major glycoprotein; *smco3*, single-pass membrane and coiled-coil domain-containing protein 3; *ccl5*, C-C motif chemokine 5; *ifi44*, interferon-induced protein 44; *cldn5*, claudin-5 Transmembrane protein deleted in VCFS; *hspb1*, heat shock protein beta-1; *vegfr1* (*flt1*), vascular endothelial growth factor receptor 1; *ctgf*, connective tissue growth factor CCN family member 2.

**Figure S5. Identification of FMO and UGT gene families.** (a) Maximum likelihood tree (TBLASTN (with *e*-value of 1×10^-5^)) of FMO gene family. Different colors represent different FMO gene families and different branch colors represent different species. (b) Maximum likelihood tree (TBLASTN (with *e*-value of 1×10^-5^)) of UGT gene family. Different colors represent different UGT gene families and different inner circle colors represent different species

**Figure S6. qPCR validation of RNA-seq data** **from the drug stress trial.**

**Figure S7. The expression levels of *ugt* genes in liver tissues of the loach under five drug stress.** Mis115330.1 and Mis0135560.1 (*ugt2a2*), UDP-glucuronosyltransferase 2A2; Mis0072610.1 (*ugt2a1*), UDP-glucuronosyltransferase 2A1.

**Figure S8. Tissue expression analysis and the knockout of *fmo5* (ID: Mis0185930.1) gene (*fmo5*^-/-^) in loach *Misgurnus anguillicaudatus* genome.** (a) Tissue expression analysis of *fmo5* (ID: Mis0185930.1) of the loach. (b) Schematic position of the CRISPR/Cas9 target site for *fmo5* gene knockout. (c) The mRNA and protein expression levels of fmo5 in livers of wild-type (WT) and *fmo5*^-/-^ loach. Different letters above error bars indicate significant difference among different tissues (*p* < 0.05); *** extremely significant difference (*p* < 0.001).

**Figure S9. Survival rates of wild-type (WT) and *fmo5*deletion (*fmo5*^-/-^) loach under five drug stress.**

**
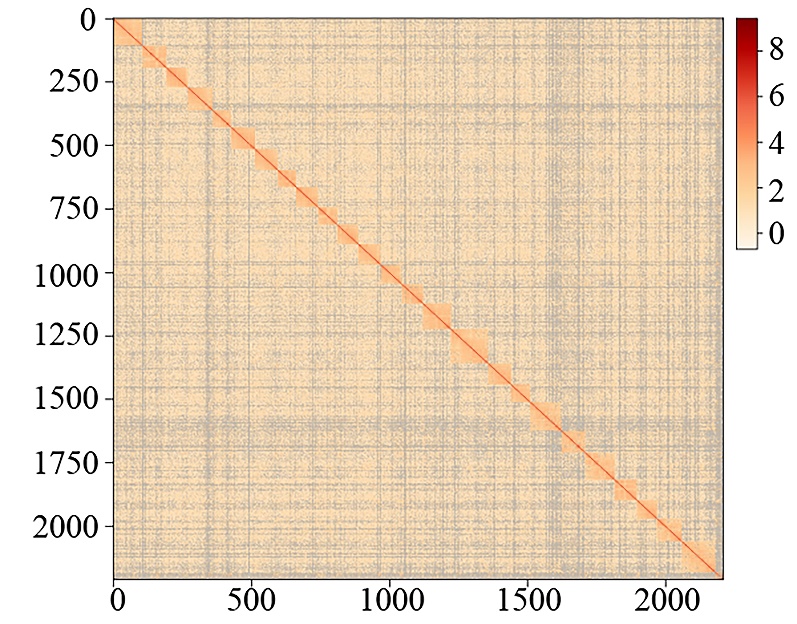
**

**Fig S1. Genome-wide Hi-C interaction map**.

**
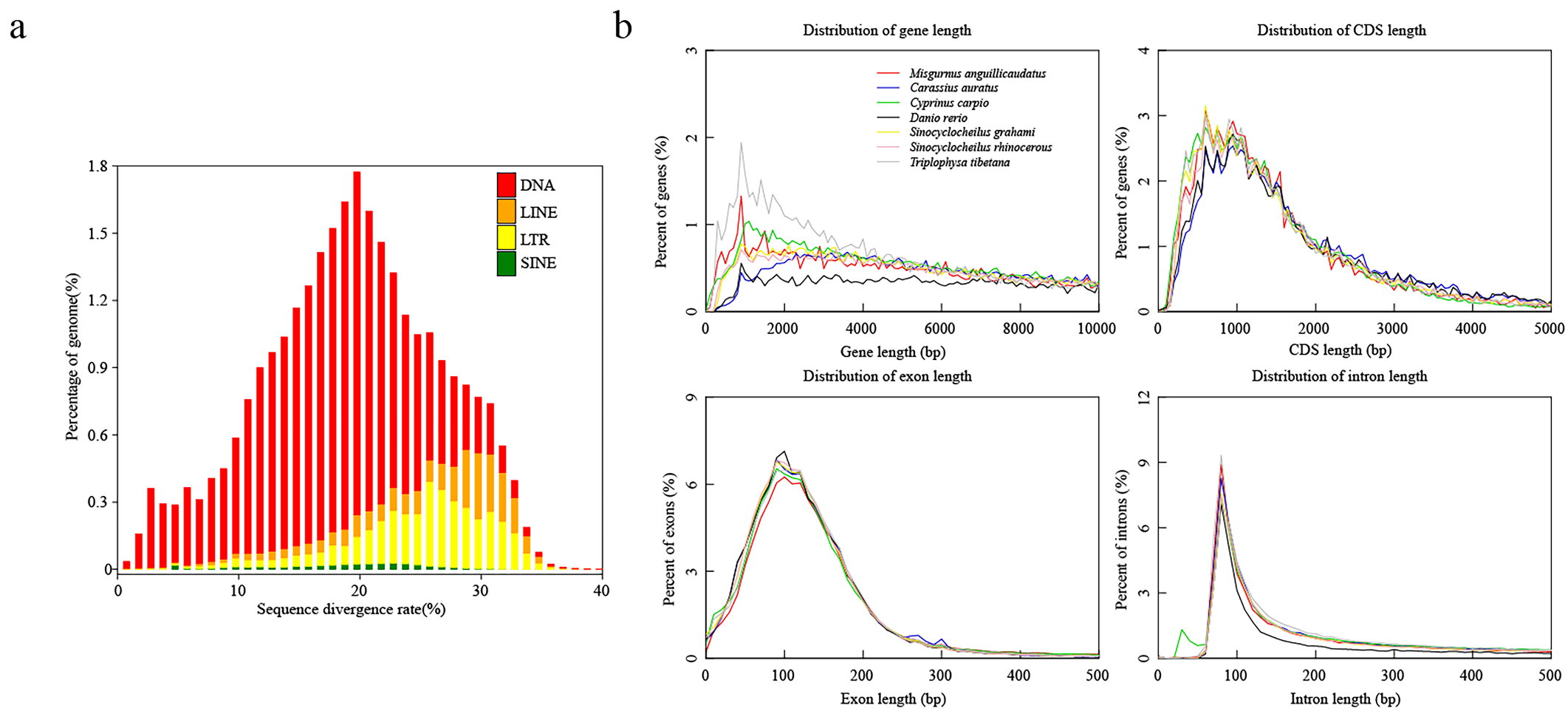
**

**Fig S2. Divergence distributions of four TE sequences predicted by the *de novo* method (a) and comparison of the distribution of several features in the final gene set for seven fish species (b).** TE, transport elements; DNA, DNA transposons; LTR, long terminal repeats; LINE, long interspersed nuclear elements; SINE, short interspersed nuclear element. The seven fish species: *Misgurnus anguillicaudatus*, *Carassius auratus*, *Cyprinus carpio*, *Danio rerio*, *Sinocyclocheilus graham*, *S. rhinocerous* and *Triplophysa tibetana*.


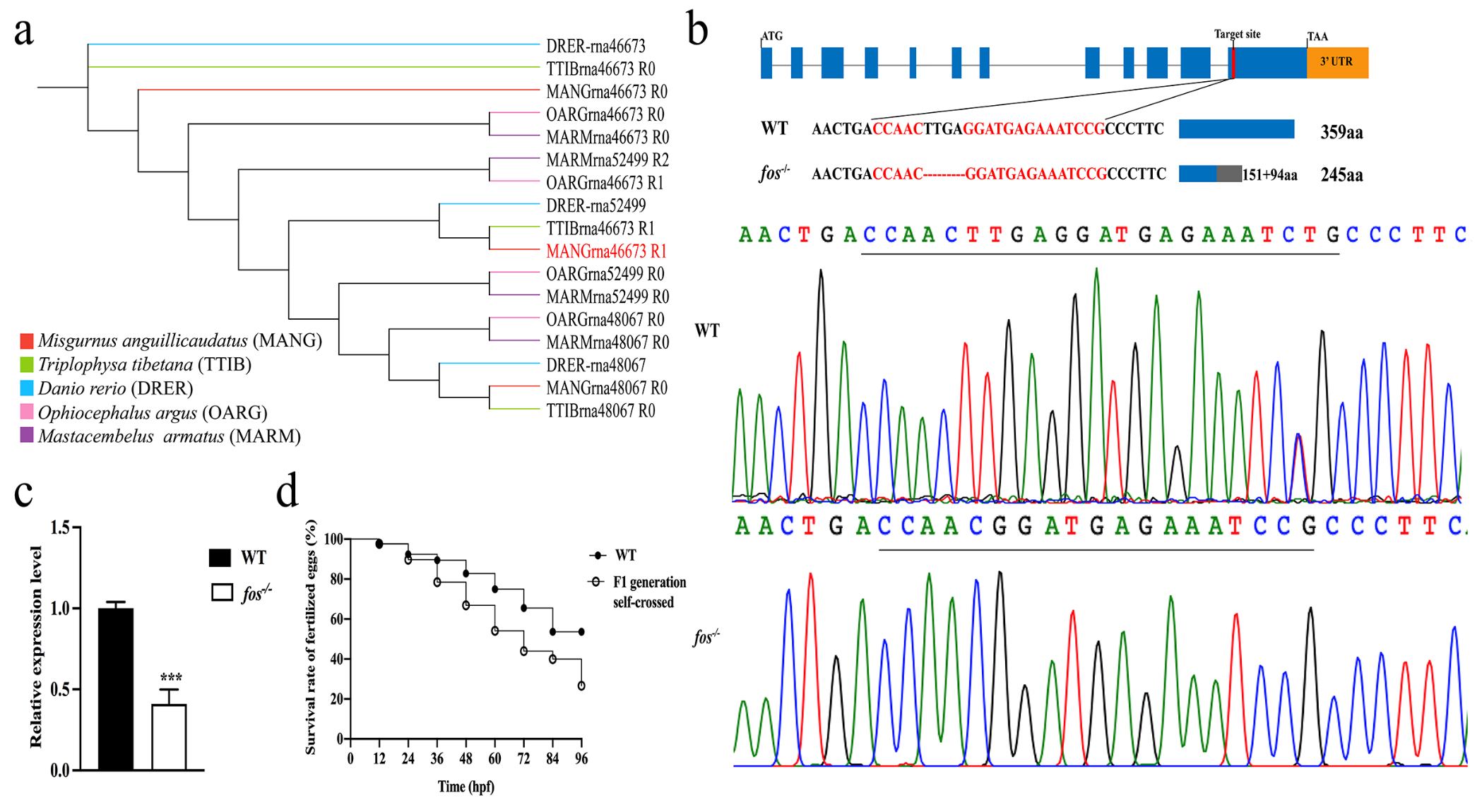


**Fig S3. The construction of maximum likelihood tree of *fos* gene and generation of the *fos* (ID: Mis0086400.1) knockout loach (*fos^-/-^*) by CRISPR/Cas9 technology.** (a) Maximum likelihood tree (TBLASTN (with *e*-value of 1×10^-5^)) of *fos* gene in fish species. Different branch colors represent different species. Red words indicate the knockout *fos* gene in loach genome. **(**b) Schematic position of the CRISPR/Cas9 target site for *fos* gene knockout. (c) The transcription level of *fos* in livers of wild-type loach (WT) and *fos^-/-^* loach. (d) A statistics of survival rate of fertilized eggs of WT loach and heterozygous F1 generation (F1 generation self-crossed). *** extremely significant difference (*p* < 0.001). hpf, hours post fertilization; *fos*, Proto-oncogene c-Fos. The gene in red color is a positively selected gene in the loach genome.


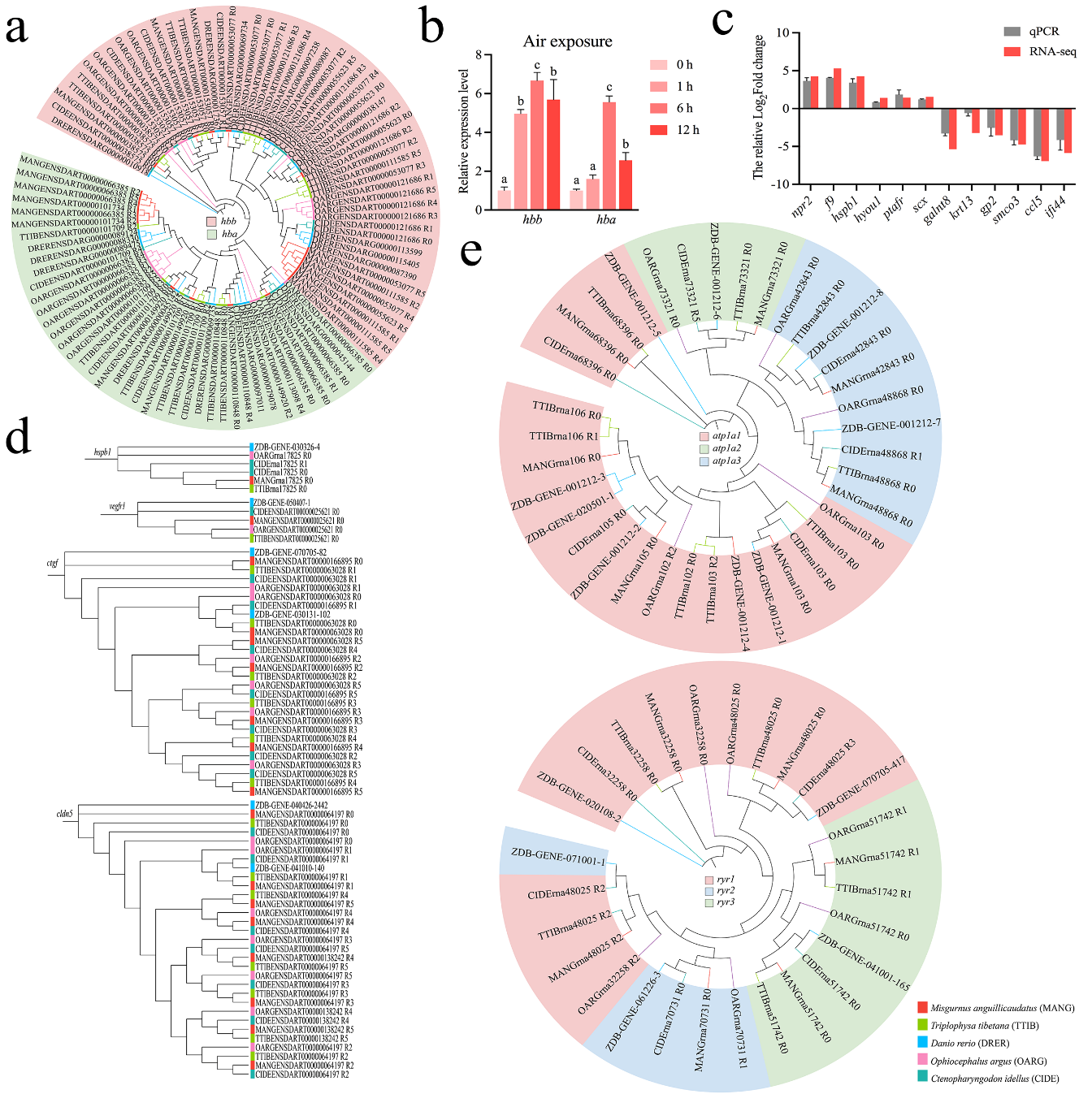


**Fig S4. Identification and expression analysis of air-breathing- and digestion/absorption- related genes in loach *Misgurnus anguillicaudatus*.** (a) Maximum likelihood tree (TBLASTN (with *e*-value of 1×10^-5^)) of *hb* gene family. Different color backgrounds represent different genes. Different branch colors and inner circle colors represent different species. *hbb*, hemoglobin subunit beta; *hba*, hemoglobin subunit alpha. (b) Expression levels of *hbb* and *hba* in posterior intestines of the loach under air exposure. (c) qPCR validation of RNA-seq data of loach posterior intestines under air exposure. (d) Maximum likelihood tree (TBLASTN (with *e*-value of 1×10^-5^)) of air-breathing-related genes. Different branch colors represent different species. (e) Maximum likelihood tree of digestion/absorption-related genes (TBLASTN (with *e*-value of 1×10^-5^)). *Atp1a*, sodium/potassium-transporting ATPase subunit; *ryr*, ryanodine receptor. Different color backgrounds represent different genes. Different branch colors represent different species. *Npr2*, atrial natriuretic peptide receptor 2; *f9*, coagulation factor IX; *hspb1*, heat shock protein beta-1; *hyou1*, hypoxia up-regulated protein 1; *ptafr*, platelet-activating factor receptor; *scx*, basic helix-loop-helix transcription factor scleraxis; *galnt8*, polypeptide N-acetylgalactosaminyltransferase 8; *krt13*, keratin, type I cytoskeletal 13 Cytokeratin-13; *gp2*, pancreatic secretory granule membrane major glycoprotein; *smco3*, single-pass membrane and coiled-coil domain-containing protein 3; *ccl5*, C-C motif chemokine 5; *ifi44*, interferon-induced protein 44; *cldn5*, claudin-5 Transmembrane protein deleted in VCFS; *hspb1*, heat shock protein beta-1; *vegfr1* (*flt1*), vascular endothelial growth factor receptor 1; *ctgf*, connective tissue growth factor CCN family member 2.


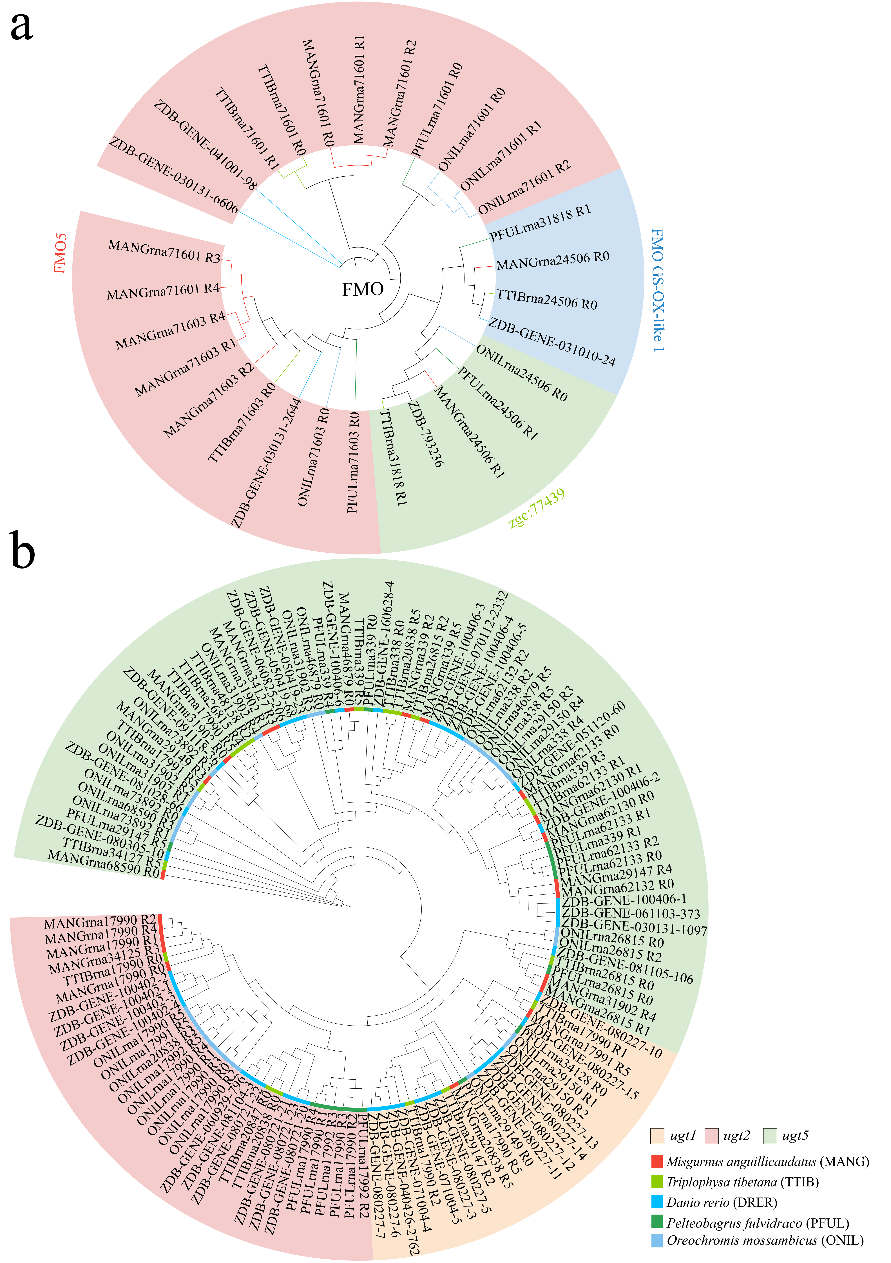


**Fig S5. Identification of FMO and UGT gene families.** (a) Maximum likelihood tree (TBLASTN (with *e*-value of 1×10^-5^)) of FMO gene family. Different colors represent different FMO gene families and different branch colors represent different species. (b) Maximum likelihood tree (TBLASTN (with *e*-value of 1×10^-5^)) of UGT gene family. Different colors represent different UGT gene families and different inner circle colors represent different species.


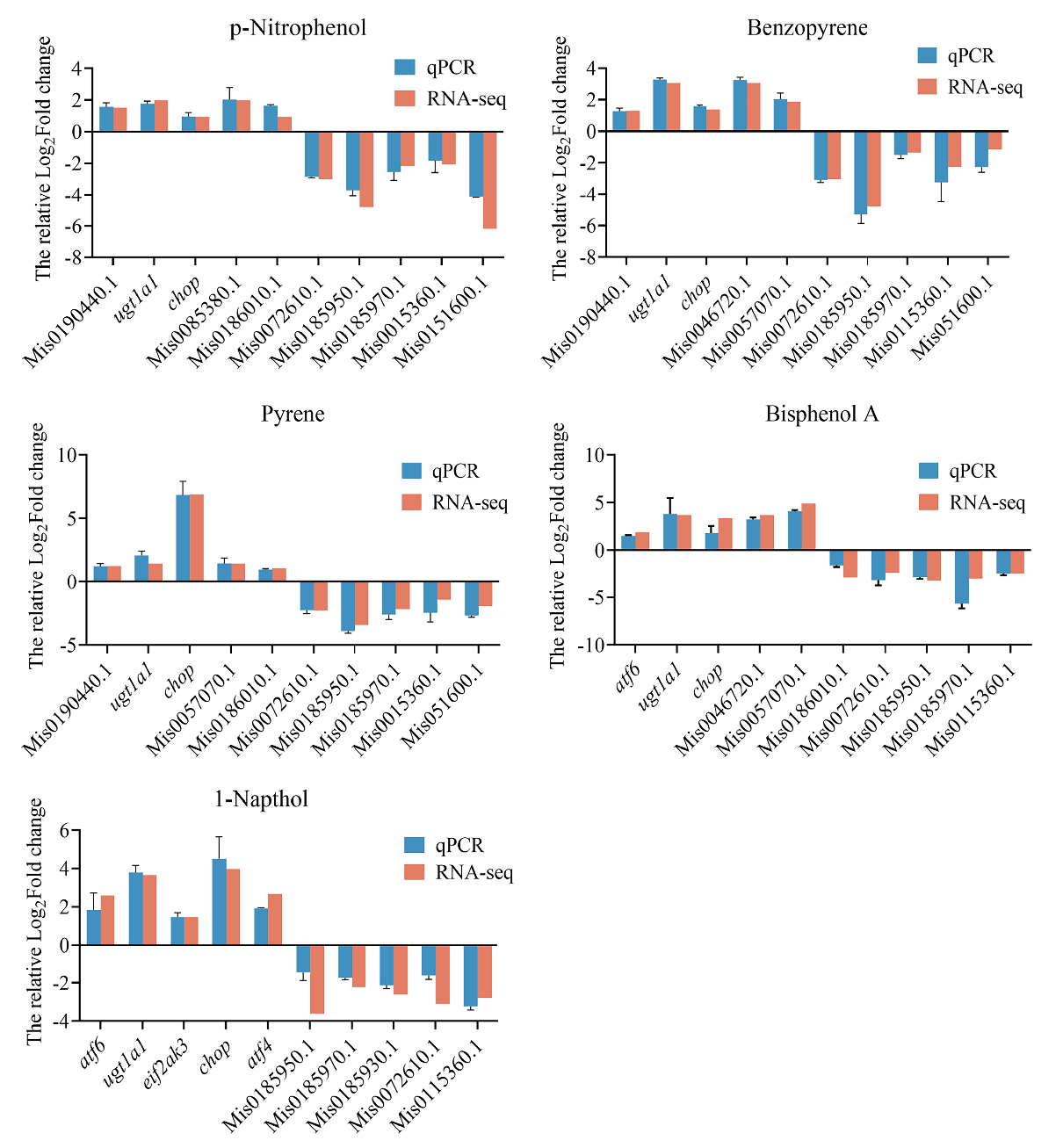


**Fig S6. qPCR validation of RNA-seq data** **from the drug stress trial.** *atf6*, cyclic AMP-dependent transcription factor; *eif2ak3*, eukaryotic translation initiation factor 2-alpha kinase 3; *chop*, DNA damage-inducible transcript 3 protein; *muc2*, mucin-2; Mis0185950.1, Mis0185940.1, Mis0185920.1, Mis0185970.1, Mis0186000.1, Mis0186010.1 (*fmo5*), dimethylaniline monooxygenase [N-oxide-forming] 5; Mis0115330.1, Mis0115330.1, Mis0135560.1 (*ugt2a2*), UDP-glucuronosyltransferase 2A2; Mis0072610.1 (*ugt2a1*), UDP-glucuronosyltransferase 2A1; *atf4*, cyclic AMP-dependent transcription factor 4; *ugt1a1*, UDP-glucuronosyltransferase 1-1; Mis0085380.1 (*cdc40*) Pre-mRNA-processing factor 17 Cell division cycle 40 homolog PRP17 homolog; Mis0151600.1 (*epha8*) Ephrin type-A receptor 8; Mis0046720.1 (*ugt1a1*) UDP-glucuronosyltransferase 1-1; Mis0057070.1 (*gsto1*) Glutathione S-transferase omega-1; Mis0190440.1 (*ppp4r3b*) Serine/threonine-protein phosphatase 4 regulatory subunit 3.


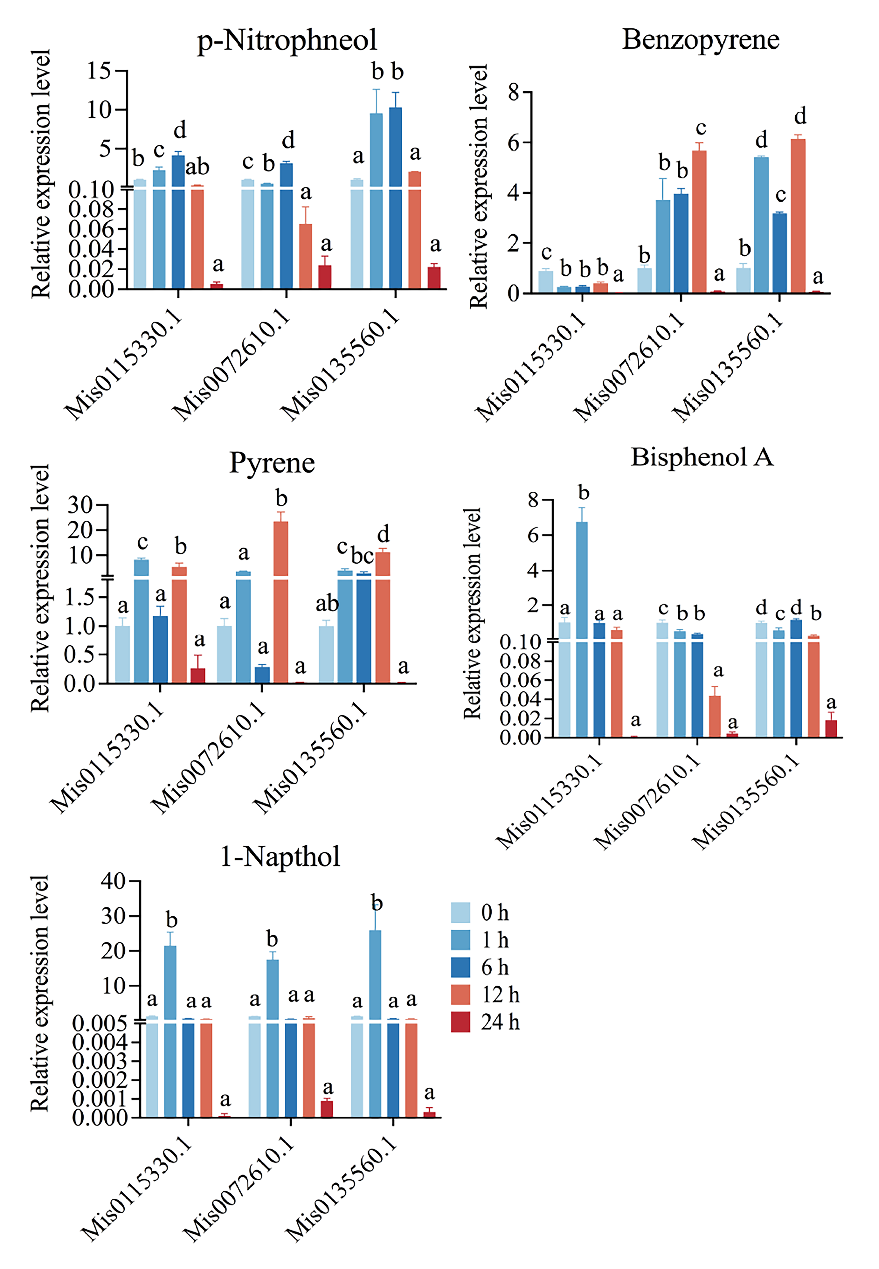


**Fig S7. The expression levels of *ugt* genes in liver tissues of the loach under five drug stress.** Mis115330.1 and Mis0135560.1 (*ugt2a2*), UDP-glucuronosyltransferase 2A2; Mis0072610.1 (*ugt2a1*), UDP-glucuronosyltransferase 2A1.


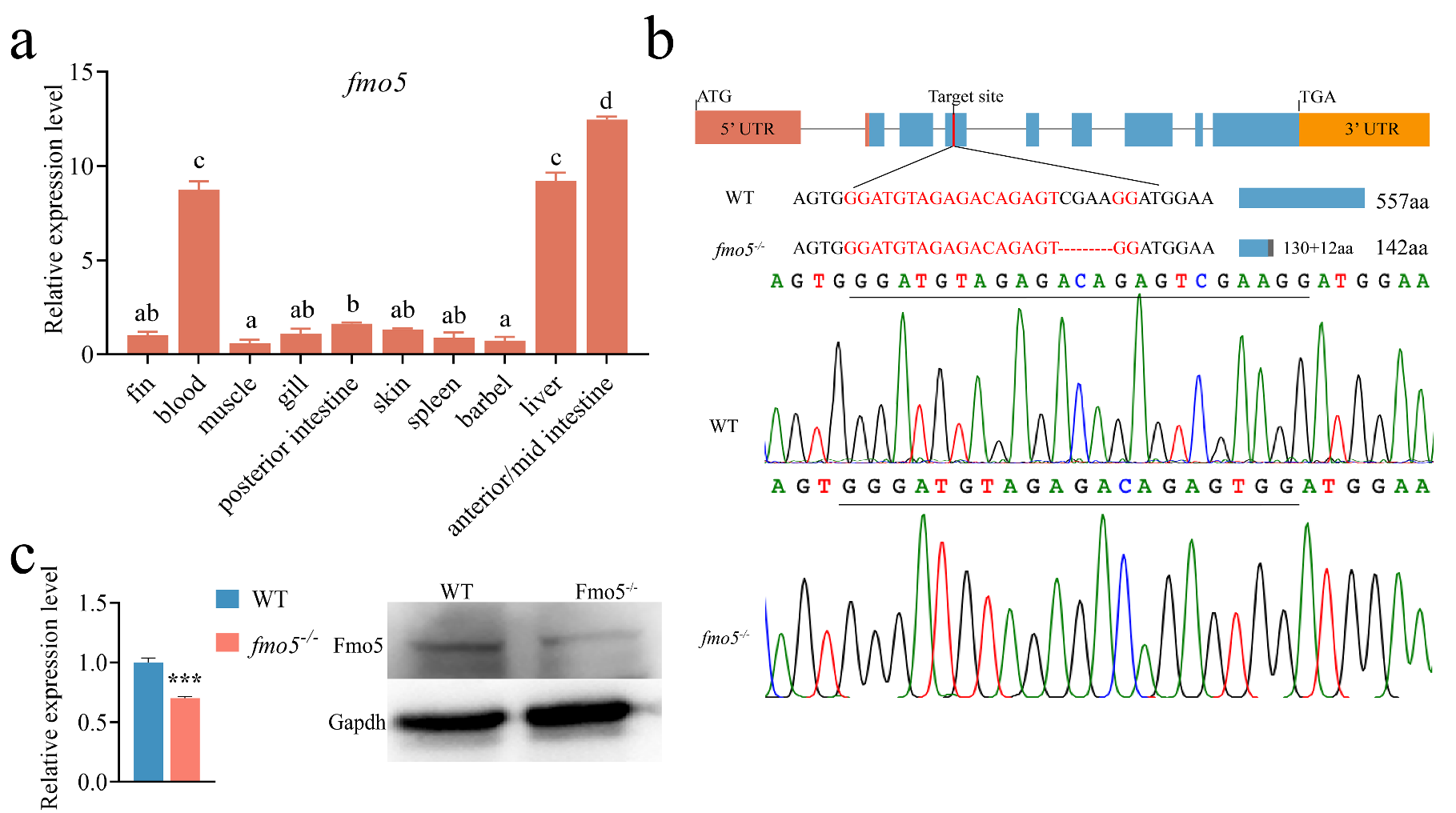


**Fig S8. Tissue expression analysis and the knockout of *fmo5* (ID: Mis0185930.1) gene (*fmo5^-/-^*) in loach *Misgurnus anguillicaudatus* genome.** (a) Tissue expression analysis of *fmo5* (ID: Mis0185930.1) of the loach. (b) Schematic position of the CRISPR/Cas9 target site for *fmo5* gene knockout. (c) The mRNA and protein expression levels of *fmo5* in livers of wild-type (WT) and *fmo5^-/-^* loach. Different letters above error bars indicate significant difference among different tissues (*p* < 0.05); *** extremely significant difference (*p* < 0.001).

**
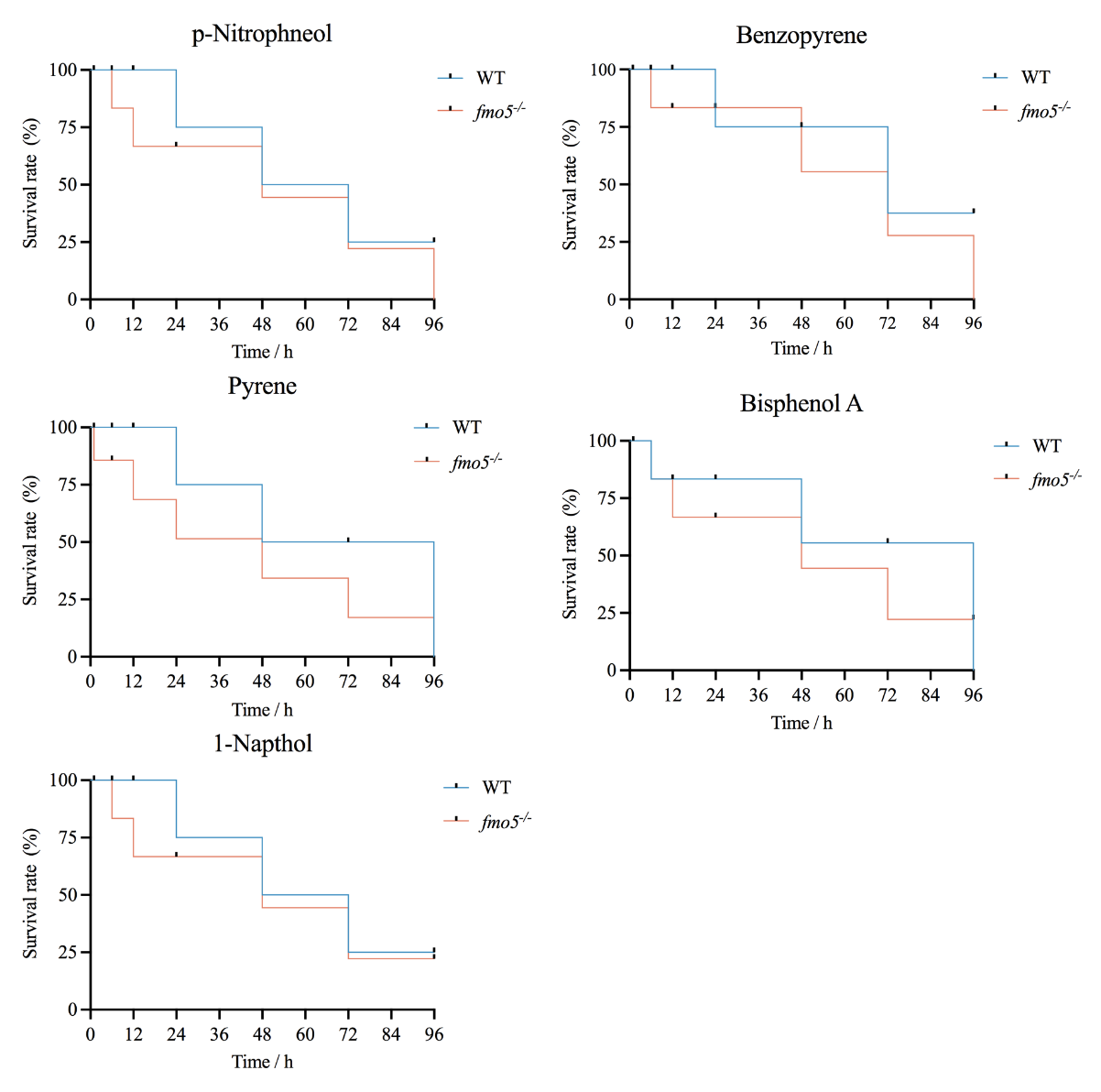
**

**Fig S9. Survival rates of wild-type (WT) and *fmo5* deletion (*fmo5^-/-^*) loach under five drug stress.**
